# An International, Multicentered, Evidence-Based Reappraisal of Genes Reported to Cause Congenital Long QT Syndrome

**DOI:** 10.1161/CIRCULATIONAHA.119.043132

**Published:** 2020-01-27

**Authors:** Arnon Adler, Valeria Novelli, Ahmad S. Amin, Emanuela Abiusi, Melanie Care, Eline A. Nannenberg, Harriet Feilotter, Simona Amenta, Daniela Mazza, Hennie Bikker, Amy C. Sturm, John Garcia, Michael J. Ackerman, Raymond E. Hershberger, Marco V. Perez, Wojciech Zareba, James S. Ware, Arthur A.M. Wilde, Michael H. Gollob

**Affiliations:** 1 Division of Cardiology, Toronto General Hospital and University of Toronto, Canada (A.A, M.C., M.H.G.).; 2 Fondazione Policlinico Universitario A. Gemelli Istituto di Ricovero e Cura a Carattere Scientifico, and Istituto di Medicina Genomica, Università Cattolica del Sacro Cuore, Rome, Italy (V.N., E.A., S.A., D.M.).; 3 Heart Center, Department of Clinical and Experimental Cardiology, Amsterdam Cardiovascular Sciences (A.S.A., A.A.M.W.), Amsterdam University Medical Centers, University of Amsterdam, The Netherlands.; 4 Department of Clinical Genetics (E.A.N., H.B.), Amsterdam University Medical Centers, University of Amsterdam, The Netherlands.; 5 Department of Pathology and Molecular Medicine, Queen’s University, Kingston, Canada (H.F.).; 6 Geisinger Genomic Medicine Institute, Danville, PA (A.C.S.).; 7 Invitae Corporation, San Francisco, CA (J.G.).; 8 Departments of Cardiovascular Diseases, Pediatrics, and Molecular Pharmacology and Experimental Therapeutics, Divisions of Heart Rhythm Services and Pediatric Cardiology, Windland Smith Rice Sudden Death Genomics Laboratory, Rochester, MN (M.J.A.).; 9 Divisions of Human Genetics and Cardiovascular Medicine in the Department of Internal Medicine, Ohio State University, Columbus (R.E.H.).; 10 Division of Cardiovascular Medicine, Department of Medicine, Stanford University, CA (M.V.P.).; 11 Cardiology Unit of the Department of Medicine, University of Rochester Medical Center, NY (W.Z.).; 12 National Heart and Lung Institute and Medical Research Council London Institute of Medical Sciences, Imperial College London, UK (J.S.W.).; 13 Royal Brompton and Harefield Hospitals National Health Service Trust, London, UK (J.S.W.).; 14 Columbia University Irving Medical Center, New York (A.A.M.W.).; 15 Department of Physiology, University of Toronto, and The Toronto General Hospital Research Institute, University Health Network, University of Toronto, Canada (M.H.G.).

**Keywords:** ClinGen, genetics, long QT syndrome, sudden death

## Abstract

**Background:**

Long QT syndrome (LQTS) is the first described and most common inherited arrhythmia. Over the last 25 years, multiple genes have been reported to cause this condition and are routinely tested in patients. Because of dramatic changes in our understanding of human genetic variation, reappraisal of reported genetic causes for LQTS is required.

**Methods:**

Utilizing an evidence-based framework, 3 gene curation teams blinded to each other’s work scored the level of evidence for 17 genes reported to cause LQTS. A Clinical Domain Channelopathy Working Group provided a final classification of these genes for causation of LQTS after assessment of the evidence scored by the independent curation teams.

**Results:**

Of 17 genes reported as being causative for LQTS, 9 (*AKAP9, ANK2, CAV3, KCNE1, KCNE2, KCNJ2, KCNJ5, SCN4B, SNTA1*) were classified as having limited or disputed evidence as LQTS-causative genes. Only 3 genes (*KCNQ1, KCNH2, SCN5A*) were curated as definitive genes for typical LQTS. Another 4 genes (*CALM1, CALM2, CALM3, TRDN*) were found to have strong or definitive evidence for causality in LQTS with atypical features, including neonatal atrioventricular block. The remaining gene (*CACNA1C*) had moderate level evidence for causing LQTS.

**Conclusions:**

More than half of the genes reported as causing LQTS have limited or disputed evidence to support their disease causation. Genetic variants in these genes should not be used for clinical decision-making, unless accompanied by new and sufficient genetic evidence. The findings of insufficient evidence to support gene-disease associations may extend to other disciplines of medicine and warrants a contemporary evidence-based evaluation for previously reported disease-causing genes to ensure their appropriate use in precision medicine.

## Abstract 2

Supplemental Digital Content is available in the text.

**Clinical Perspective**

**What Is New?**

- Of 17 genes previously reported as causing long QT syndrome (LQTS), 9 were found to have limited or disputed evidence for disease causation.
- Only 3 genes were found to have definitive evidence for causation of typical LQTS.
- Four genes are definitively associated with LQTS with atypical features that can include QT prolongation associated with neonatal heart block or autosomal-recessive inheritance.

**What Are the Clinical Implications?**

- Genes with limited or disputed evidence for disease causation should not be routinely tested in the evaluation of patients and families with LQTS, as interpretation of any identified variants cannot appropriately be classified in relation to disease.
- Genetic testing for genes (*CALM1, CALM2, CALM3, TRDN*) in LQTS with atypical features should be considered in patients with clinical observations concordant with the specific phenotypic expression demonstrated in reported cases.

**Editorial, see p 440**

In 1957, Jervell and Lange-Nielsen described the first cases of autosomal-recessive long QT syndrome (LQTS) with concomitant bilateral sensorineural deafness, providing the first description of an inherited arrhythmia syndrome associated with sudden cardiac death in structurally normal hearts.^1^ Subsequently, in 1963 and 1964,^2,3^ Drs Romano and Ward described the autosomal-dominant version of LQTS with an isolated cardiac phenotype. Over 3 decades later, in 1995, the first genes thought to underlie the pathophysiology of LQTS were discovered by Mark Keating’s research team.^4,5^ Their identification of rare genetic variants in the *KCNH2*-encoded Kv11.1 potassium channel and the *SCN5A*-encoded Nav1.5 sodium channel in families with LQTS was revolutionary in our understanding of not only this condition but also all inherited arrhythmia syndromes. The concept of a pathogenic genetic variant in a gene encoding a cardiac ion channel leading to a highly arrhythmogenic substrate has become the blueprint for the pathophysiology of all of these syndromes and has made LQTS the prototype of the “channelopathies.” These seminal discoveries also propelled a genetic research race for identification of other genes associated with LQTS. Two and a half decades later, the list of genes reported to cause LQTS has grown to include 17 genes.^6^

These findings have made it possible for genetic testing to become a routine part of the evaluation of patients suspected of having LQTS.^7,8^ Identified pathogenic genetic variants may then be used for genetic screening of family members, which facilitates diagnosis and risk stratification of relatives and implementation of therapies to reduce sudden cardiac death risk. Because sudden cardiac death may be the first presentation of LQTS and because it is the most common inherited arrhythmia syndrome, estimated to affect 1:2000 individuals,^9^ the clinical impact of genetic testing for this syndrome is especially high. At the same time, incorrect interpretation of genetic information can lead to harmful results.^10,11^ Diagnosing healthy individuals as having LQTS based on misinterpreted genetic findings can cause undue anxiety, unnecessary lifestyle changes, insurance or occupation-related adverse impacts, and in some cases inappropriate medical interventions such as implantable-cardioverter-defibrillator placement.

Whereas the early 2000’s witnessed an exponential surge in reported gene causes of human disease because of the success of the Human Genome Project, in more recent years our understanding of the natural variation within the human genome has significantly evolved. Advancing technology leading to reduced costs for genomic sequencing has facilitated the sequencing of large reference populations such as the Exome Sequencing Project^12^ and the genome aggregation database (gnomAD),^13^ which includes data from over 140,000 individuals. This enormous amount of information has led to important new insights regarding the spectrum of human genetic variation. We now know that some variants that were thought to be rare are in fact common in populations, and that rare genetic variants are collectively extremely common, with the majority of rare variants not causative for Mendelian disease.^14^ It is in this context that the National Institutes of Health initiated the development of the Clinical Genome Resource (ClinGen), a resource comprised of an international consortium of clinicians, geneticists, genetic counselors and researchers that aims to define the clinical relevance of genes and genetic variants in the application of precision medicine for patient care.^11,15^ With this goal in mind, a standardized, evidence-based framework for assessment of reported gene-disease associations has been developed.^16,17^ Herein, we report the results of the International, Multicentered LQTS ClinGen Working Group implementing this framework for a reappraisal of all previously published LQTS-causative genes.

## Methods

All supporting data are available within the article and its files in the online-only Data Supplement.

Selection of genes for evaluation by the Working Group was performed by a PubMed search including the terms “long QT,” “gene,” and “genetic” limited to publications in the English language and to human studies until January 2017: [Long qt[title] AND ((“genes”[MeSH Terms] OR “genes”[All Fields] OR “gene”[All Fields]) OR (“genetic therapy”[MeSH Terms] OR (“genetic”[All Fields] AND “therapy”[All Fields]) OR “genetic therapy”[All Fields] OR “genetic”[All Fields])) AND ((“0001/01/01”[PDAT]: “2017/01/31”[PDAT]) AND “humans”[MeSH Terms] AND English[lang])]. Publications were screened as required for identification of genes reported to be involved in causality of congenital LQTS. This effort did not evaluate the validity of any gene for disorders other than LQTS.

Three gene curation teams were formed to independently curate each gene, as previously described.^15^ Gene curation teams were comprised of 3 members per team, and worked blinded to other curation teams in applying the ClinGen Gene Curation Framework.^16^ Curation team members were required to review a Standard Operating Procedure for gene curation using this framework^18^ and received training in the application of the analytic process. The framework provides a systematic, evidence-based approach for assessing reported gene-disease associations. Using a semiquantitative scoring system, each gene-disease relationship is categorized into a clinical validity classification level (Definitive, Strong, Moderate, Limited) based on the sum of its accompanying evidence. Clinical validity classification levels include the following: Definitive (12–18 points and replicated literature), Strong (12–18 points), Moderate (7–11 points), and Limited (1–6 points). Briefly, the evidence-based framework evaluates genetic and experimental data separately and provides a scoring metric based on the level of evidence provided in the published literature for the gene. Clinical evidence supporting the phenotype of disease, in this case LQTS, is not evaluated and assumed to be accurately reported based on standard clinical criteria (eg, QTc, Schwartz score). Genetic evidence scores were weighted according to the design and quality of the genetics study. For example, a gene implicated in a study with familial data, variant-disease segregation and comprehensive sequencing of a linked genomic region would receive a greater assigned score than a gene implicated through a “candidate gene” approach evaluating a small cohort of cases only and without an adequate control group. Experimental evidence scores were based on the interpretation and phenotypic relevance of in vitro assays assessing functional alterations of the disease-implicated gene variants, and model organism or rescue studies, as proposed by MacArthur et al.^19^

A Clinical Domain Working Group (https://www.clinicalgenome.org/affiliation/40025/), consisting of 11 additional individuals with collectively dozens of years of experience in the clinical care and research of LQTS, was tasked with reviewing the 3 independent classifications, performing a synthesized evaluation and assigning a final classification on a gene-by-gene basis. This panel had the option of modifying the classification of each gene (upgrade, no change, downgrade) after review of curator team summaries. A classification of Disputed reflects the panel’s conclusion that there is an absence of any substantive evidence to support a gene’s association for causality of LQTS.

Each individual on the group independently reviewed data for each gene, and together the group discussed classifications for each gene in teleconference and face-to-face meetings to reach a final consensus of the gene’s classification for disease causation.

Websites of commercial genetic laboratories performing LQTS-specific genetic panels and registered in the National Center for Biotechnology Information’s Genetic Testing Registry (https://www.ncbi.nlm.nih.gov/gtr/) were searched for lists of genes included in panels (accessed in February to March 2019). Panels including conditions other than LQTS (eg, “arrhythmia” panels) and those limited to single genes were excluded.

## Results

### Summary of Findings

**Figure 1.**
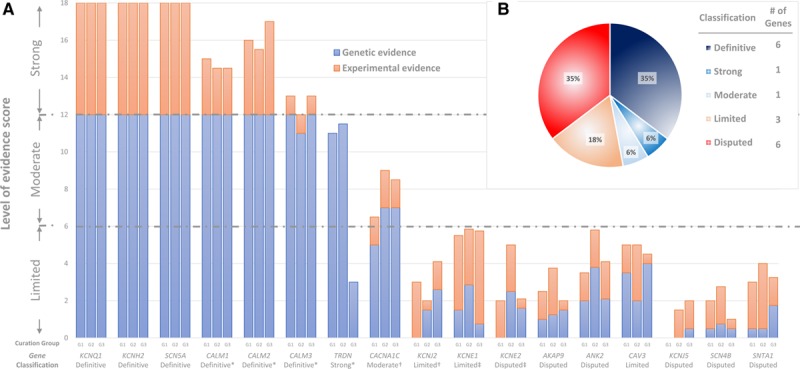
**Disease causality classification and level of evidence scores for genes reported in LQTS**. **A**, Validity scores according to the ClinGen curation framework and final classifications of the Working Group. Genetic and experimental evidence scores of each one of the 3 blinded curating teams are detailed. Complete list of references used for scoring of these genes is available in the supplements. **B**, Distribution of genes according to classification. *Genes with strong or definitive evidence for causality in LQTS with atypical features. †Genes with definitive evidence for causality in inherited multiorgan syndrome including QT prolongation but only moderate or limited evidence for isolated LQTS. ‡Genes with strong level of evidence for causality in acquired LQTS but only limited or disputed evidence for congenital LQTS.

A PubMed literature search identified 17 genes reported to cause LQTS in humans (Table 1). Of these, the ClinGen Working Group classified more than half (9/17) as having either disputed evidence (6) or limited evidence (3) for disease causation (Table 2 and Figure 1). Only 3 of 17 genes, *KCNQ1*, *KCNH2*, and *SCN5A*, were classified as having definitive evidence as a genetic cause for typical LQTS. Four other genes, *CALM1*, *CALM2*, *CALM3*, and *TRDN*, had definitive or strong evidence supporting their role in causing LQTS, but with specific atypical features (see Genes With Strong or Definitive Evidence for Disease Causality). The final gene, *CACNA1C*, was graded as having moderate evidence for disease causation.

**Table 1.**
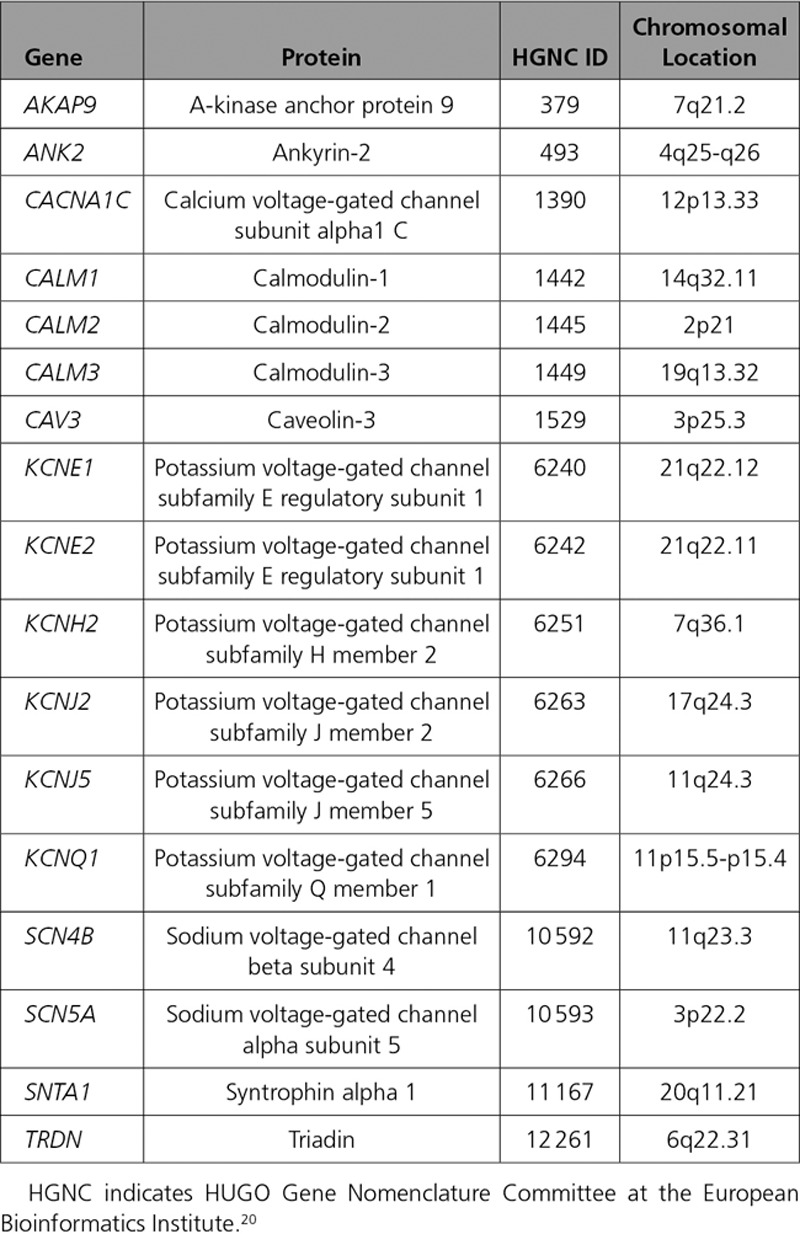
Reported Genes for Long QT Syndrome

**Table 2.**
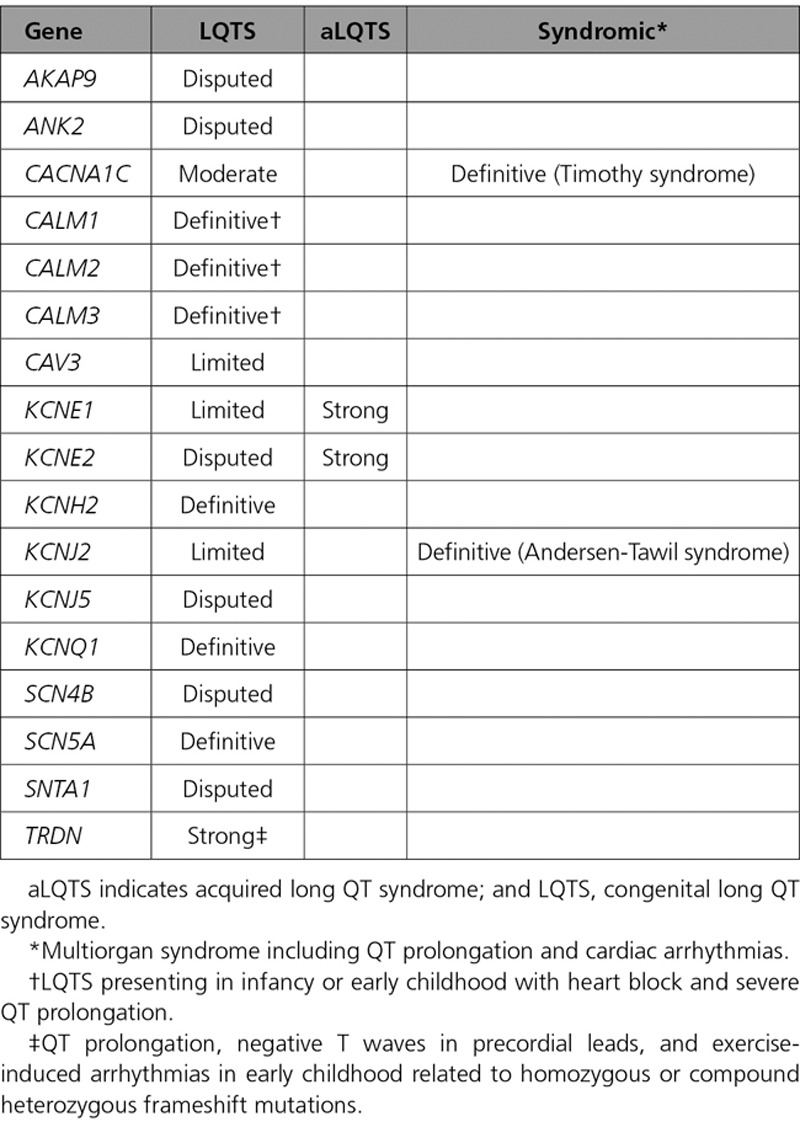
Classification of Genetic Evidence for Genes Previously Reported as Causing LQTS

During the reappraisal process, the Working Group identified 2 genes, *KCNE1* and *KCNE2*, as having numerous reports supporting their role in the etiology of drug or electrolyte-provoked LQTS, referred to as acquired LQTS (aLQTS), in addition to being reported as causes of congenital LQTS. The Working Group therefore decided to classify these 2 genes separately for aLQTS and LQTS. Because the ClinGen framework was not constructed for analysis of genes with risk alleles, classification of evidence for association with aLQTS was based on available curated data and the expert opinion of the Working Group. Although *KCNE1* was classified as having only limited evidence and *KCNE2* as having disputed evidence for causality in LQTS, both genes were classified as having strong evidence for specific risk alleles in predisposing to aLQTS (Table I in the online-only Data Supplement).

Two genes, *CACNA1C* and *KCNJ2*, were reported to be associated with multiple organ system involvement (Timothy Syndrome and Andersen-Tawil Syndrome, respectively), which include a prolonged QT interval and ventricular arrhythmias as part of the phenotypic expression. The Working Group classified these genes separately for their role in the full multiorgan syndromes and for their role for causing only the cardiac-specific phenotype of LQTS. Although both genes were classified as having definitive evidence for the multiorgan syndromes, the level of evidence for the cardiac-specific phenotype was classified as only moderate for *CACNA1C* and limited for *KCNJ2.*

### Disputed Genes and Genes With Limited Evidence for Disease Causality

Six genes, *AKAP9, ANK2, KCNE2, KCNJ5, SCN4B*, and *SNTA1*, were classified as disputed, defined as the absence of sufficient genetic evidence to support causation of LQTS (Figure 1). Publications on 4 of these genes (*AKAP9, KCNE2, SCN4B, SNTA1*) were based on a candidate gene approach, in contrast to an unbiased genome-wide methodology, and lacked evidence of statistically significant segregation of the suspected variant in multiple affected cases beyond chance alone. Further literature, including case-control studies, to extend the level of genetic evidence to support causality for these genes was absent. For 2 genes, *ANK2* and *KCNJ5*, the initial publication was based on a linkage analysis in large families.^21,22^ Although such an approach is unbiased in localizing a shared genomic region harboring a significantly smaller proportion of candidate genes shared among affected cases, both studies implicating these genes had significant limitations. In the case of *ANK2*, the linked genomic region on chromosome 4 was reported to encompass close to 16 million base pairs, incorporating dozens of genes not comprehensively evaluated.^21,23^ Importantly, the identified genetic variant reported in *ANK2*, p.Glu1458Gly, is now known to have a population frequency too high to be an autosomal-dominant cause of LQTS, and is observed in approximately 1/650 individuals of European descent and 1/400 individuals of Latino ancestry.^24^ Similarly, *KCNJ5* was within a 16 million base pair–linked region (11q23.3-24.3) in a large Chinese family,^22^ and the reported Gly387Arg variant is now recognized to be present in 1/200 individuals of East Asian descent.^25^ The Working Group concluded that according to contemporary knowledge and standards, the available evidence is insufficient to demonstrate disease-gene association and that the genetic variants identified in these genes are more likely to represent normal human genetic variation.

Three genes, *CAV3, KCNE1*, and *KCNJ2*, were classified as having limited evidence to support an etiologic role in LQTS (Figure 1). Genetic evidence associating these genes with disease causality was also based on a candidate gene approach and was limited in scope. Nevertheless, for each of these 3 genes, there was additional evidence leading the Working Group to conclude they have some, albeit limited, evidence for gene-disease association. Specifically, this included the reported observation of de novo variants in *CAV3* cases, although parental phenotype and confirmation of paternity was not specifically stated in the report.^26^ *KCNJ2* is a gene with definitive evidence for causation of Andersen-Tawil syndrome, a condition associated with ECGs manifesting prominent U waves often interpreted as QT prolongation. Because there are other examples of syndromes impacting multiple organs that may also present with isolated cardiac findings (eg, Fabry disease^27^), the evidence in Andersen-Tawil syndrome was thought to lend some support to gene-disease association in isolated LQTS. Similarly, the strong evidence for a role of *KCNE1* in aLQTS (file in the online-only Data Supplement) led the panel to classify it as having limited evidence for disease causality for unprovoked LQTS, although studies in large families with variant segregation is lacking. Furthermore, several case reports have identified homozygous or compound heterozygous rare variants in *KCNE1* in patients with Jervell and Lange-Nielsen syndrome; however, parents or siblings carrying only 1 allele have reported normal phenotypes,^28–30^ suggesting an association of this gene with an autosomal-recessive form of LQTS. Although *KCNE2* was concluded to have strong evidence for aLQTS, a recent comprehensive review of reported *KCNE2* variants reported for LQTS demonstrated that *KCNE2* variants routinely require secondary provocation to induce phenotype, persuading the Working Group to conclude that *KCNE2* has no supportive evidence as a cause of LQTS in the absence of provoking factors.^31^

### Genes With Strong or Definitive Evidence for Disease Causality

For the 3 genes first associated with LQTS, *KCNH2, SCN5A*, and *KCNQ1*, there was definitive evidence for a gene-disease association. For each of these genes, genetic evidence was based on linkage analysis in >1 family,^4,5,32^ and was supported by an abundance of genetic and experimental evidence accumulated over decades of research and clinical observations.

**Figure 2.**
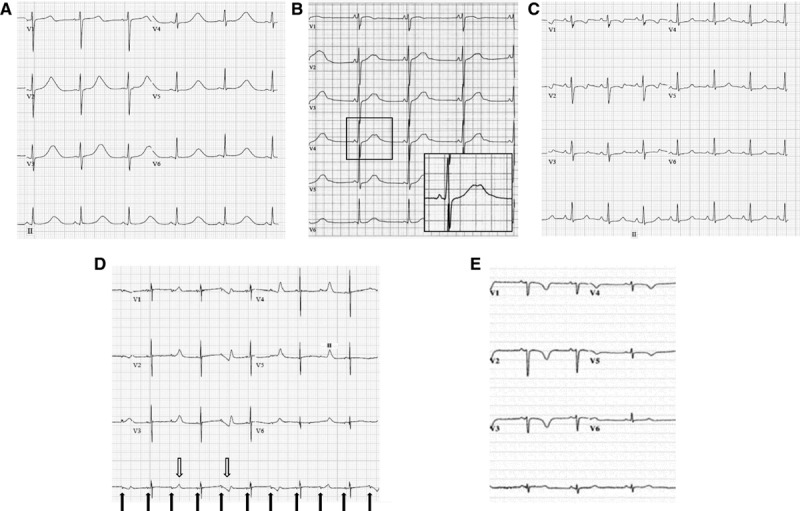
**ECGs of typical and atypical LQTS.** ECGs of typical broad-based T waves in long QT 1 (**A**), bifid T waves in long QT 2 (**B**, enlarged box), and long horizontal ST-segment in long QT 3 (**C**). **D**, ECG of a 1-day-old infant found to carry a de novo variant in *CALM3* (c.389A>G, p.Asp130Gly). A very prolonged QT interval with 2:1 atrioventricular block (full arrows) and T-wave alternans (hollow arrows) is present. Adapted with permission from Reed et al.^39^ **E**, An ECG demonstrating QT prolongation and negative T waves in precordial leads of a 10-year-old girl homozygous for a loss-of-function variant in *TRDN* (c.53_56delACAG, p.Asp18Alafs). Adapted with permission from Altmann et al.^41^

The 3 calmodulin genes (*CALM1-3*) were scored separately but discussed as a group because they all encode for an identical protein.^33^ *CALM1* and *CALM2* were first associated with LQTS by exome sequencing of 2 unrelated infants with QT prolongation where de novo variants were identified.^34^ Subsequent studies uncovered further genetic evidence for these genes, including *CALM3.*^35–39^ Most recently, the International Calmodulinopthy Registry has published a series of 36 LQTS cases secondary to rare variants distributed among all 3 CALM genes.^40^ In all cases identified, no other family members were found to be phenotype-positive and were thus concordant with de novo variants, although not in all families was this proven by sequencing of both parents and confirmation of paternity. Furthermore, the clinical characteristics of these cases were similar and included presentation in infancy or early childhood (up to 5 years) with marked bradycardia or atrioventricular block associated with severe QT prolongation, a presentation that is seen only rarely in LQTS related to *SCN5A* and *KCNH2* genetic defects (Figure 2).^34,36–39^ In view of the abundance of data supporting de novo variants for all 3 *CALM* genes, and in concordance with the scoring provided by independent curation groups, the Working Group concluded a classification of Definitive for the calmodulin genes (*CALM1-3*) for disease causation of LQTS with atypical features presenting in childhood.

Evidence for involvement of *TRDN* in LQTS was based mainly on a single publication demonstrating 5 cases with homozygous or compound heterozygous frameshift variants.^41^ All cases presented during early childhood (up to the age of 3 years) with QT prolongation, negative T waves in precordial leads (Figure 2), and exercise-induced arrhythmias, although typical torsades de pointes was demonstrated only in 1 case. Experimental evidence demonstrated that *TRDN* loss of function may lead to arrhythmogenesis but did not specifically show prolongation of repolarization, which is the hallmark of LQTS. Accordingly, there was a debate within the panel as to whether the *TRDN*-related cardiac phenotype should be classified as catecholaminergic polymorphic ventricular tachycardia or as a unique syndrome, referred in the literature as triadin knockout syndrome.^42^ Because QT prolongation was the most easily discernable abnormality, it was decided to consider these cases as having an atypical LQTS phenotype. Furthermore, it was agreed that there was strong evidence for *TRDN*’s disease association.

### Analysis of Commercial LQTS Genetic Panels

**Figure 3.**
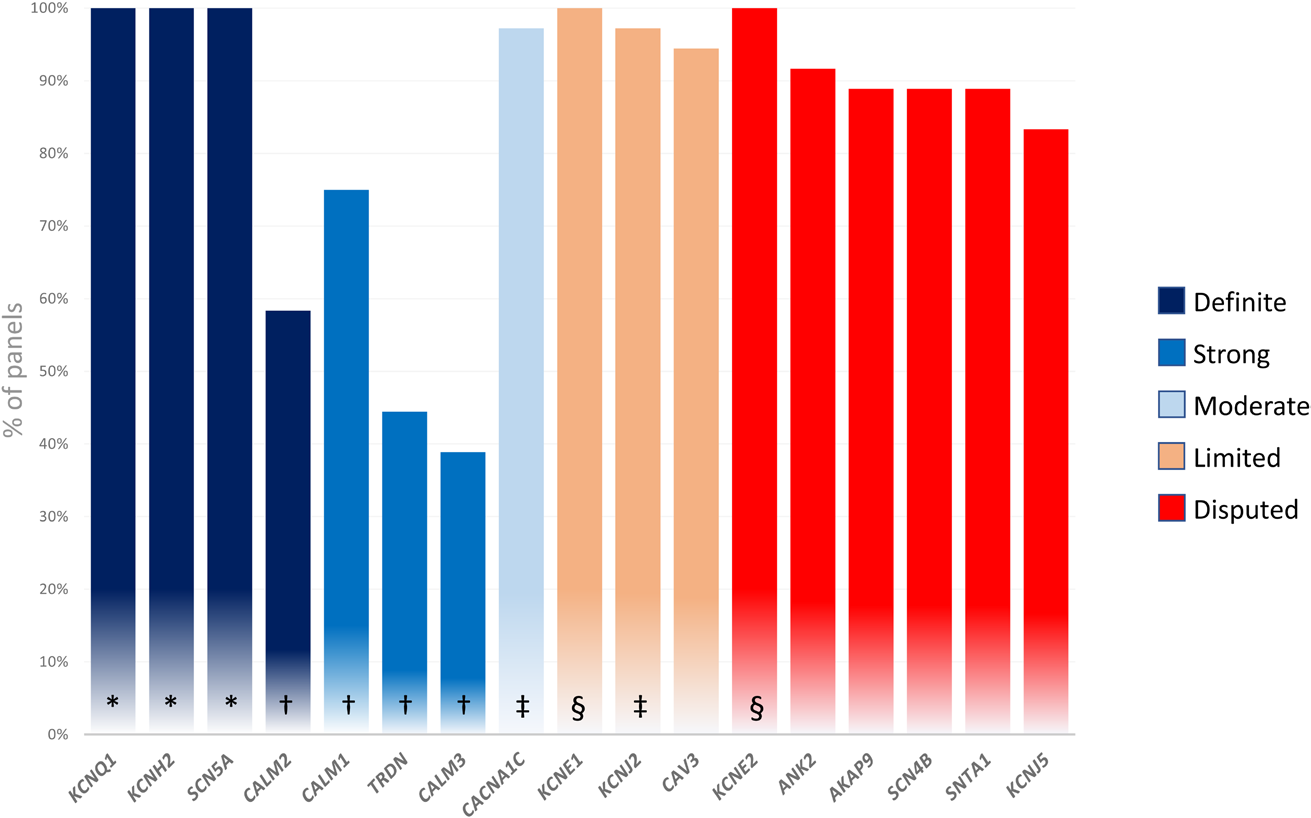
**Composition of LQTS-specific genetic panels.** Percentage of genetic panels including genes previously reported as causing LQTS. *Genes with definitive evidence for causality of typical LQTS. †Genes with strong or definitive evidence for causality in LQTS with atypical features. ‡Genes with definitive evidence for causality in multiorgan including QT prolongation but only moderate or limited evidence for isolated LQTS. §Genes with strong level of evidence for causality in acquired LQTS but only limited or disputed evidence for congenital LQTS.

LQTS-specific genetic panels of 36 genetic laboratories (20 from North America, 13 from Europe, and 3 from Australia/New Zealand—see the detailed list in the online-only Data Supplement) included 5 to 17 of the genes previously reported as causing LQTS. Only *KCNQ1, KCNH2, SCN5A, KCNE1*, and *KCNE2* were included in every commercial panel (Figure 3). Genes classified as having definitive or strong evidence for causality of LQTS with atypical features (*CALM 1, CALM 2, CALM3, TRDN*) were least likely to be included and were found in only 39% to 75% of panels. Genes reported as disputed or with limited evidence for LQTS were routinely tested in 83% to 100% of the panels.

## Discussion

LQTS is the most common inherited arrhythmia syndrome and the prototype channelopathy. Its clinical evaluation involves assessment of both electrocardiographic and genetic information, the latter of which is now generally obtained through gene sequencing panels offered by commercial genetic testing laboratories. The current study utilized the evidence-based ClinGen framework^16^ for reappraisal of the genes previously reported as causing LQTS. Out of 17 genes evaluated, more than half were classified as disputed or having limited evidence for disease causation. Independently, curation teams demonstrated a high degree of uniformity in applying the evidence-based classification matrix and in reaching concordant final scores. Similarly, a final review of curated data by the Clinical Domain Working Group resulted in complete agreement with curator team conclusions and near unanimous consensus on final gene classifications, highlighting the effectiveness of this evidence-based methodology.

Classifications of genes as disputed or limited were based on a combination of the following reasons: (1) use of a candidate gene approach in the seminal discovery studies, which is more likely to yield false-positive results in the absence of an unbiased genome-wide gene discovery method; (2) identification of variants that were subsequently found to be relatively common in certain populations, making them unlikely culprits for an uncommon and potentially lethal disease such as LQTS; (3) lack of data demonstrating segregation of the identified variants with manifest prolonged QT in sufficiently sized familial studies; or (4) absence of data supporting statistically significant excess of rare variants in cases as compared with controls.

Interestingly, 2 genes (*ANK2, KCNJ5*) were initially reported following a linkage analysis approach. Although such an unbiased genome-wide approach for gene discovery is preferable to a hypothesis-driven candidate gene approach that excludes consideration of the remainder of the genome, limitations do exist. For diseases that may have diagnostic challenges, such as LQTS, incorporating inconclusive cases or disease phenocopies as “affected status” in linkage modeling may significantly impact the accuracy of the identified linked genomic region. Importantly, linked regions may also be large in size and contain dozens to hundreds of genes, and include other genes not yet identified. Specific to the linked regions reported for *ANK2* and *KCNJ5*, these regions contained dozens of genes not evaluated.^21,22^ The most compelling reason, however, leading to classification of these genes as disputed arises from the relatively high frequency of the reported variants in contemporary large population databases, data which were unavailable during the publication of the seminal discovery papers.

Only 3 genes (*KCNQ1, KCNH2*, and *SCN5A*) were classified as having definitive evidence supporting their causality in typical LQTS. Four other genes (*TRDN* and *CALM1-3*) were classified as having strong or definitive evidence supporting an etiology for LQTS with atypical features. The final gene studied, *CACNA1C*, was found to have a definitive association with Timothy syndrome but only moderate evidence supporting a cardiac-only phenotype concordant with LQTS.

Taken as a whole, this contemporary, evidence-based evaluation of reported LQTS disease-genes challenges the classic concept of the genetic landscape in LQTS according to which there are 3 “major” genes responsible for 75% to 95%^8,43^ of cases and a myriad of “minor” genes that are each responsible for a small fraction of the LQTS patient population. The current reappraisal portrays a vastly different landscape with only 3 genes (*KCNQ1, KCNH2*, and *SCN5A*) causing typical LQTS and another 4 genes (*CALM1-3* and *TRDN*) responsible for rare cases of infantile/pediatric LQTS with atypical features. Although it is probable that the missing heritability in LQTS is due, at least in part, to rare single pathogenic variants in genes as yet unidentified, this hypothesis is still to be confirmed. Alternatively, non-Mendelian inheritance (eg, oligogenic) or phenocopies may be responsible for some of these gene-elusive cases.

### Genes Associated With LQTS With Atypical Features

The 3 *CALM* genes, located on different chromosomes (Table 1), all encode for the identical protein, calmodulin, which is involved in many calcium-dependent intracellular processes, including regulation of ionic channels.^34^ The Working Group classified all 3 CALM genes as having definitive evidence for causation of LQTS. It was noted that published cases with *CALM* rare genetic variants had specific and atypical features. These included proven or suspected de novo variants and presentation at infancy or early childhood (up to 5 years) with marked sinus bradycardia or atrioventricular block, QT prolongation, seizures, and developmental delay.

Triadin is another protein involved in calcium-dependent processes in cardiomyocytes including regulation of calcium release and excitation-contraction coupling.^41^ The Working Group classified *TRDN* as having strong evidence for causality of atypical LQTS. Atypical features demonstrated by all identified cases included autosomal-recessive inheritance, presentation in infancy or early childhood (up to 3 years), and negative T waves in precordial leads. Variants in *CALM1-3* or *TRDN* should be suspected in patients with these clinical presentations.

Presently, *CALM1-3* and *TRDN* are present in only 40% to 75% of commercial genetic panels (Figure 3). The role of these genes in causing more typical presentations of LQTS remains to be established, particularly in adult cases.

### Genes Associated With Multiorgan Syndromes Including QT Prolongation

Andersen-Tawil syndrome (*KCNJ2*) and Timothy syndrome (*CACNA1C*) are rare conditions affecting multiple systems whose phenotypic expression includes QT prolongation and ventricular arrhythmias. In Andersen-Tawil syndrome, extracardiac manifestations include dysmorphic features and periodic paralysis with hypo- and hyperkalemic episodes in some patients.^44^ The cardiac manifestation includes QT-U abnormalities but not typical QT prolongation.^45^ Ventricular arrhythmias also differ from typical LQTS with frequent premature ventricular complexes and polymorphic nonsustained ventricular tachycardia but only rarely torsades de pointes. In Timothy syndrome, multiple systems may be involved, including dysmorphic facial features, developmental delay, endocrine abnormalities, and congenital heart defects.^46^ Electrophysiological abnormalities include bradycardia, atrioventricular block, QT prolongation, and polymorphic ventricular arrhythmias.^46^

The rationale behind using these genes as candidates in LQTS in reported studies relied on precedent in which some multiple-organ syndromes may present with a cardiac-specific phenotype in isolation (eg, Fabry disease).^27^ Nevertheless, the Working Group found only limited evidence for *KCNJ2* as a disease-causing gene in isolated LQTS. Although curator teams and the Working Group agreed that *CACNA1C* had some evidence to support its possible role as a cause of isolated LQTS, the limited published human genetic data at the present time resulted in a consensus for this gene as having moderate-level evidence for isolated LQTS. As such, extra care should be utilized before classifying variants in these genes as pathogenic when found in cases suspicious of LQTS.

### Genes Associated With aLQTS

Genetic variants in genes associated with aLQTS are thought to predispose carriers to the development of QT prolongation and torsades de pointes in the presence of other QT-prolonging factors (eg, medications, electrolyte abnormalities). These genetic variants cause relatively minor perturbations in ionic currents that are insufficient by themselves for the development of LQTS. It is, however, reasonable to hypothesize that some variants in these genes have a greater effect. It is for this reason that *KCNE1* and *KCNE2*, which have been associated with aLQTS,^31,47^ were studied as candidate genes in LQTS. Although both genes were classified by the Working Group as having strong evidence for causation in aLQTS, there was a paucity of evidence supporting causality for LQTS. Indeed, Roberts et al reached similar conclusions in their analysis of *KCNE2*, finding no evidence for a LQTS phenotype in the absence of other QT-prolonging factors in patients carrying rare or uncommon variants in this gene.^31^ One possible exception is evidence from several case reports suggesting an association between rare variants in *KCNE1* and autosomal-recessive Jervell and Lange-Nielsen syndrome.^28–30^ In such rare cases, homozygous or compound heterozygous rare *KCNE1* variants should be evaluated carefully for disease causality. It should be noted in this context that relatively common genetic variants in other genes (eg, *NOS1AP*), not known to be single-gene causes of LQTS, have been described as having a mild impact on ionic currents and have been associated with mild QT prolongation in population studies and with severity of phenotype in LQTS.^48,49^ Curation of such risk alleles or polygenic risk scores was not part of this ClinGen Working Group’s mandate, requires a different analytical process, and are not included in this study.

### Clinical Implications

The guidelines of the American College of Medical Genetics and Genomics for interpretation of genetic variants explicitly warn against their utilization in genes of unproven significance.^50^ Accordingly, their use for interpretation of variants in the genes with insufficient evidence (Limited, Disputed) to support a causal role in LQTS should be discouraged. Nevertheless, ClinVar currently contains variants classified as pathogenic or likely pathogenic in these disputed and limited evidence genes. More frequently, variants in disputed genes are classified as variants of uncertain significance. This too, however, holds a potential for unwanted outcomes as it may lead to uncertainty for clinicians, counselors, and patients and unnecessary expenses or anxieties if family members are screened for these variants.

The current study also reflects on the commonly used nomenclature in LQTS. Because of their typical genotype-phenotype associations, LQTS attributable to pathogenic variants in *KCNQ1, KCNH2*, and *SCN5A* have long been categorized as LQTS1-3.^7,9^ This nomenclature has been extended to LQTS4-17 as further genes associated with LQTS were reported. In view of our findings demonstrating many of these reported genes as having disputed or limited evidence for disease causation, it may be appropriate to limit the use of numbered LQTS to LQTS1-3 and the rest to their pathogenic basis, such as *CALM1*-LQTS rather than LQT14. Last, the application of precision medicine in clinical care requires the accurate and appropriate use of genetic testing to optimize the care of patients and families. Our study provides specific insights into the contemporary understanding of the genetic architecture underlying LQTS and should facilitate the provision of appropriate genetic testing panels offered to patients and families with suspected LQTS.

### Implications for Future Research

The classifications of gene-disease association in this manuscript may be used as a guide for what genetic and experimental evidence may be impactful in the future. For instance, case reports of patients with LQTS with variants in definitive genes would be of limited added value. However, segregation studies or studies demonstrating excess of rare variants in cases versus controls would be most useful for establishing stronger gene-disease association in genes with lower levels of evidence.

The fact that even in LQTS, the prototype of channelopathies and one of the most studied genetic heart diseases, contemporary reevaluation asserts that most previously reported gene-disease associations are based on disputed or limited data highlights the importance of curating gene, variant, and disease-level data according to present-day knowledge. Such curation endeavors are important for improving clinical care and provide a more robust foundation for future research of inherited conditions and the clinical application of precision medicine.

## Acknowledgments

M.H.G. designed the study. A.A., M.C., H.F., A.S.A., E.A.N., H.B., E.A., S.A., and D.M. performed the evidence-based gene curation. J.G., M.C., A.C.S., V.N., J.S.W., R.E.H., M.J.A., W.Z., M.V.P., A.A.M.W., and M.H.G. comprised the National Institutes of Health-Channelopathy Clinical Domain Working Group and reviewed the curation data and analyses.

A.A. summarized the data and generated figures. M.H.G. and A.A. wrote the article. All authors reviewed and edited the article. The authors thank Lisa Kurtz, PhD, for her assistance in the curation process.

## Sources of Funding

The US National Human Genome Research Institute partially funded this study. Funding agency members had no role in the study design or collection, analysis, and interpretation of the data or writing of the manuscript. The corresponding author had full access to all the data in the study and had final responsibility for the decision to submit for publication. The Clinical Genome Resource Consortium is funded by the National Human Genome Research Institute (U41 HG006834, U01 HG007436). Dr Ware is supported by the Wellcome Trust (107469/Z15/Z), the Medical Research Council (United Kingdom), and the National Institute for Health Research Imperial Biomedical Research Centre. Dr Ackerman is supported by the Mayo Clinic Windland Smith Rice Comprehensive Sudden Cardiac Death in the Young Program. Dr Wilde is supported by The Netherlands Cardiovascular Research Initiative (Predict). Dr Gollob is supported by the Canadian Institutes for Health Research (408226).

## Disclosures

Dr Ackerman is a consultant for Audentes Therapeutics, Boston Scientific, Gilead Sciences, Invitae, Medtronic, MyoKardia, and St Jude Medical. Dr Ackerman and the Mayo Clinic have a potential equity/royalty relationship with AliveCor. However, none of these entities participated in this study. The other authors report no conflicts.

## Supplementary Material


