# Supplementary material for: An International, Multicentered, Evidence-Based Reappraisal of Genes Reported to Cause Congenital Long QT Syndrome
Source: Circulation. 2020 Jan 27;141(6):418–28. doi: 10.1161/CIRCULATIONAHA.119.043132 (PMC7017940; doi:10.1161/CIRCULATIONAHA.119.043132)
Supplement: Supplementary file 1 [file cir-141-418-s001.pdf]

Supplemental material

List of investigators:

NIH-ClinGen Clinical Domain Working Group:

Michael H Gollob, M.D. (Chair)
The Division of Cardiology, Toronto General Hospital and The Toronto General Hospital Research Institute, University Health Network and University of Toronto, Toronto, ON, Canada

Michael Ackerman, M.D. Ph.D
Departments of Cardiovascular Diseases, Pediatrics, and Molecular Pharmacology and Experimental Therapeutics, Divisions of Heart Rhythm Services and Pediatric Cardiology, Windland Smith Rice Sudden Death Genomics Laboratory, Rochester, MN

John Garcia, Ph.D.
Invitae Corp. San Francisco, CA, USA

Ray E Hershberger. M.D.
Divisions of Human Genetics and Cardiovascular Medicine in the Department of Internal Medicine, Ohio State University, Columbus, OH, USA

Valeria Novelli, Ph.D.
Fondazione Policlinico Universitario A. Gemelli I.R.C.C.S., and Istituto di Medicina Genomica, Università Cattolica del Sacro Cuore, Rome, Italy

Marco V Perez. M.D.
Division of Cardiovascular Medicine, Department of Medicine, Stanford University, Stanford, CA, USA

Amy C Sturm, Ms.C
Geisinger Genomic Medicine Institute, Danville, PA, USA

James S Ware, Ph.D., M.R.C.P.
National Heart & Lung Institute and MRC London Institute of Medical Sciences, Imperial College London, Royal Brompton and Harefield Hospitals NHS Trust, London, UK

Arthur AAM Wilde. M.D. Ph.D,
Department of Clinical and Experimental Cardiology, Amsterdam Cardiovascular Sciences, Amsterdam UMC, University of Amsterdam, Heart Center; Amsterdam, The Netherlands

Wojciech Zareba. M.D., Ph.D
Cardiology Unit of the Department of Medicine, University of Rochester Medical Center, Rochester, NY, USA

Bio-curators:

Emanuela Abiusi, Ph.D
Fondazione Policlinico Universitario A. Gemelli I.R.C.C.S., and Istituto di Medicina Genomica, Università Cattolica del Sacro Cuore, Rome, Italy

Arnon Adler, M.D.
The Division of Cardiology, Toronto General Hospital, University Health Network and University of Toronto, Toronto, ON, Canada

Simona Amenta, M.D.
Fondazione Policlinico Universitario A. Gemelli I.R.C.C.S., and Istituto di Medicina Genomica, Università Cattolica del Sacro Cuore, Rome, Italy

Ahmad S Amin, M.D. Ph.D.
Department of Clinical and Experimental Cardiology, Amsterdam Cardiovascular Sciences, Amsterdam UMC, University of Amsterdam, Heart Center; Amsterdam, The Netherlands

Hennie Bikker, Ph.D.
Department of Clinical Genetics, Amsterdam UMC, University of Amsterdam, Heart Center; Amsterdam, The Netherlands

Melanie Care, Ms.C
The Division of Cardiology, Toronto General Hospital, University Health Network and University of Toronto, Toronto, ON, Canada

Harriet Feilotter, Ph.D
Department of Pathology and Molecular Medicine, Queen's University, Kingston, ON, Canada

Daniela Mazza, M.D.
Fondazione Policlinico Universitario A. Gemelli I.R.C.C.S., and Istituto di Medicina Genomica, Università Cattolica del Sacro Cuore, Rome, Italy

Eline A. Nannenberg, M.D., Ph.D.
Department of Clinical Genetics, Amsterdam UMC, University of Amsterdam, Heart Center; Amsterdam, The Netherlands

List of genetic laboratories

Admera Health (US)
Ambry (US)
Amsterdam Medical (Holland)
ASpainer Biogene (Estonia)
Bioscientia (Germany)
Blueprint (Finland)
CeGat (Germany)
Cen4Gen (Canada)
CentoGene (Germany)
CGC Genetics (Portugal)
Children's Minnesota (US)
Cincinnati Children's (US)
CTGT (US)
DDC Clinic (US)
EGL (US)
Fulgent (US)
GeneDx (US)
GeneTaq (Spain)
GPS WUSTL (US)
Greenwood (US)
Health[in]Code (Spain)
Invitae (US)
LabGenetics (Spain)
LabPlus (New Zealand)
Mayo Clinic (US)
MNG Laboratories (US)
Montreal Heart Institute (Canada)
OHSU Knight (US)
Phosphorus (US)
PreventionGenetics (US)
Reference Laboratory (Spain)
Royal Brompton (UK)
Sistemas (Spain)
Sonic (Australia)
Transgenomic (US)
VSGS (Australia)

Supplemental Table. Genetic variants in KCNE1 and KCNE2 previously reported in aLQTS cases

AA change	cDNA change	MAF (all)*	MAF† (highest)	References
KCNE1				
Asp76Asn	226G>A	0.00007	0.0001 (Eu)	[1]
Asp85Asn	253G>A	0.009	0.025 (AJ)	[1-5]
KCNE2				
Thr8Ala	22A>G	0.004	0.006 (Eu)	[2,6-8]
Gln9Glu	25C>G	0.0015	0.016 (Af)	[6]
Leu11fsTer46	Unavailable	0	0	[8]
Ile57Thr	170T>C	0.001	0.002 (Latino)	[6-8]
Met54Thr	161T>C	0.0002	0.005 (AJ)	[7]
Ala116Val	347C>T	0.00002	0.00004 (Af)	[7]
Met121Lys	362T>A	0.000008	0.00002 (Eu)	[8]

* Minor allele frequency in gnomAD

† Highest minor allele frequency in any of the sub-populations available on gnomAD: Af- African; AJ- Ashkenazi Jewish; Eu- European (non-Finnish)

aLQTS- acquired long QT syndrome; MAF- minor allele frequency

Table references

1. Weeke P, Mosley JD, Hanna D, et al. Exome sequencing implicates an increased burden of rare potassium channel variants in the risk of drug-induced long QT interval syndrome. J Am Coll Cardiol 2014;63:1430-1437.
2. Paulussen AD, Gilissen RA, Armstrong M, et al. Genetic variations of KCNQ1, KCNH2, SCN5A, KCNE1, and KCNE2 in drug-induced long QT syndrome patients. J Mol Med (Berl) 2004;82:182-188.
3. Lane CM, Giudicessi JR, Ye D, et al. Long QT syndrome type 5-Lite: Defining the clinical phenotype associated with the potentially proarrhythmic p.Asp85Asn-KCNE1 common genetic variant. Heart Rhythm 2018;15:1223-1230.

4. Kaab S, Crawford DC, Sinner MF, et al. A large candidate gene survey identifies the KCNE1 D85N polymorphism as a possible modulator of drug-induced torsades de pointes. Circ Cardiovasc Genet 2012;5:91-99.
5. Nishio Y, Makiyama T, Itoh H, et al. D85N, a KCNE1 polymorphism, is a disease-causing gene variant in long QT syndrome. J Am Coll Cardiol 2009;54:812-819.
6. Abbott GW, Sesti F, Splawski I, et al. MiRP1 forms IKr potassium channels with HERG and is associated with cardiac arrhythmia. Cell 1999;97:175-187.
7. Sesti F, Abbott GW, Wei J, et al. A common polymorphism associated with antibiotic-induced cardiac arrhythmia. Proc Natl Acad Sci U S A 2000;97:10613-10618.
8. Roberts JD, Krahn AD, Ackerman MJ, et al. Loss-of-Function KCNE2 Variants: True Monogenic Culprits of Long-QT Syndrome or Proarrhythmic Variants Requiring Secondary Provocation? Circ Arrhythm Electrophysiol 2017;10: e005282.

Gene classification matrices

AKAP9

Evidence Type					Points Counted G1	G2	G3	PMID
Genetic Evidence	Case-Level	Variant	Autosomal Dominant OR X-linked Disorder	Proband with other variant type with some evidence of gene impact	1	1.25	1.5	18093912; 28003625; 26132555; 29350269; 26189708
				Proband with predicted or proven null variant	0	0	0	
				Variant is *de novo*	0	0	0	
			Autosomal Recessive Disorder	Two variants (not predicted/proven null) with some evidence of gene impact in *trans*	0	0	0	
				Two variants in *trans* and at least one *de novo* or a predicted/proven null variant				
		Segregation		Candidate gene sequencing	0	0	0	
				Exome/genome or all genes sequenced in linkage region				
	Case-Control				0	0	0	
	**Genetic Evidence Total**				**1**	**1.25**	**1.5**	
Experimental Evidence	Functional			Biochemical functions	1	2	0.5	25037568; 11799244
				Protein interactions				18093912; 22778270; 11799244
				Expression				
	Functional Alteration			Patient cells	0.5	0.5	0	
				Non-patient cells				18093912
	Models			Non-human model organism	0	0	0	
				Cell-culture model				
	Rescue			Rescue in human				
				Rescue in non-human model organism				
				Rescue in cell culture model				
				Rescue in patient cells				
	**Experimental Evidence Total**				**1.5**	**2.5**	**0.5**	
**Total Points**					**2.5**	**3.75**	**2**	
**Summary Classification**					**Disputed**			

ANK2

Evidence Type					Points Counted G1	G2	G3	PMID
Genetic Evidence	Case-Level	Variant	Autosomal Dominant OR X-linked Disorder	Proband with other variant type with some evidence of gene impact	2	2.3	2.1	26132555;28196901;16253912;15178757;16864073;17242276;27784853;28003625
				Proband with predicted or proven null variant	0	0	0	
				Variant is *de novo*	0	0	0	
			Autosomal Recessive Disorder	Two variants (not predicted/proven null) with some evidence of gene impact in *trans*	0	0	0	
				Two variants in *trans* and at least one *de novo* or a predicted/proven null variant				
		Segregation		Candidate gene sequencing	0	0	0	12571597
				Exome/genome or all genes sequenced in linkage region				
	Case-Control				0	1.5	0	16253912;17242276;16864073
	**Genetic Evidence Total**				**2**	**3.8**	**2.1**	
Experimental Evidence	Functional			Biochemical functions	0	0	0	
				Protein interactions				
				Expression				
	Functional Alteration			Patient cells	0.5	2	2	
				Non-patient cells				28196901;15178757;17242276;10579720;28196901
	Models			Non-human model organism	1	0	0	12571597
				Cell-culture model				
	Rescue			Rescue in human				
				Rescue in non-human model organism				
				Rescue in cell culture model				
				Rescue in patient cells				
	**Experimental Evidence Total**				**1.5**	**2**	**2**	
**Total Points**					**3.5**	**5.8**	**4.1**	
**Summary Classification**					**Disputed**			

CACNA1C

Evidence Type					Points Counted G1	G2	G3	PMID
Genetic Evidence	Case-Level	Variant	Autosomal Dominant OR X-linked Disorder	Proband with other variant type with some evidence of gene impact	5	7	6	25633834;23677916;24728418;27390944
				Proband with predicted or proven null variant	0	0	0	
				Variant is *de novo*	0	0	0	15863612
			Autosomal Recessive Disorder	Two variants (not predicted/proven null) with some evidence of gene impact in *trans*	0	0	0	
				Two variants in *trans* and at least one *de novo* or a predicted/proven null variant				
		Segregation		Candidate gene sequencing	0	0	1	24728418
				Exome/genome or all genes sequenced in linkage region				23677916
	Case-Control				0	0	0	
	**Genetic Evidence Total**				**5**	**7**	**7**	
Experimental Evidence	Functional			Biochemical functions	0	0	0	
				Protein interactions				
				Expression				
	Functional Alteration			Patient cells	1.5	2	1.5	
				Non-patient cells				25633834;23677916;24728418;27390944
	Models			Non-human model organism	0	0	0	
				Cell-culture model				
	Rescue			Rescue in human				
				Rescue in non-human model organism				
				Rescue in cell culture model				
				Rescue in patient cells				
	**Experimental Evidence Total**				**1.5**	**2**	**1.5**	
**Total Points**					**6.5**	**9**	**8.5**	
**Summary Classification**					**Moderate**			

CALM1

Evidence Type					Points Counted G1	G2	G3	PMID
Genetic Evidence	Case-Level	Variant	Autosomal Dominant OR X-linked Disorder	Proband with other variant type with some evidence of gene impact	1	1	0	23388215;24076290;26969752
				Proband with predicted or proven null variant	0	0	0	
				Variant is *de novo*	12	12	12	23388215;27374306;26969752
			Autosomal Recessive Disorder	Two variants (not predicted/proven null) with some evidence of gene impact in *trans*	0	0	0	
				Two variants in *trans* and at least one *de novo* or a predicted/proven null variant				
		Segregation		Candidate gene sequencing	0	0	0.5	
				Exome/genome or all genes sequenced in linkage region				
	**Case-Control**				2	2	6	26969752;24076290
	**Genetic Evidence Total**				**12**	**12**	**12**	
Experimental Evidence	Functional			Biochemical functions	1	0.5	0.5	
				Protein interactions				16556865
				Expression				23388215
	Functional Alteration			Patient cells	2	2	2	28158429
				Non-patient cells				23388215;26969752;27374306;24958779;24816216;26309258
	Models			Non-human model organism	0	0	0	
				Cell-culture model				
	Rescue			Rescue in human				
				Rescue in non-human model organism				
				Rescue in cell culture model				
				Rescue in patient cells				
	**Experimental Evidence Total**				**3**	**2.5**	**2.5**	
**Total Points**					**15**	**14.5**	**14.5**	
**Summary Classification**					Defintive			

CALM2

Evidence Type					Points Counted G1	G2	G3	PMID
Genetic Evidence	Case-Level	Variant	Autosomal Dominant OR X-linked Disorder	Proband with other variant type with some evidence of gene impact	0	0	0	26969752
				Proband with predicted or proven null variant	0	0	0	
				Variant is *de novo*	12	12	12	24917665;23388215;27374306
			Autosomal Recessive Disorder	Two variants (not predicted/proven null) with some evidence of gene impact in *trans*	0	0	0	
				Two variants in *trans* and at least one *de novo* or a predicted/proven null variant				
		Segregation		Candidate gene sequencing	0	0	0	
				Exome/genome or all genes sequenced in linkage region				
	**Case-Control**				2	2	8	26969752
	**Genetic Evidence Total**				**12**	**12**	**12**	
Experimental Evidence	Functional			Biochemical functions	1	0.5	1	
				Protein interactions				16556865;28575668
				Expression				23388215;
	Functional Alteration			Patient cells	1	2	2	28335032;27765793
				Non-patient cells				23388215;27374306;24917665;24958779;24816216;26309258
	Models			Non-human model organism	2	1	2	
				Cell-culture model				
	Rescue			Rescue in human				
				Rescue in non-human model organism				
				Rescue in cell culture model				
				Rescue in patient cells				28335032;27765793
	**Experimental Evidence Total**				**4**	**3.5**	**5**	
**Total Points**					**16**	**15.5**	**17**	
**Summary Classification**					Definitive			

CALM3

Evidence Type					Points Counted G1	G2	G3	PMID
Genetic Evidence	Case-Level	Variant	Autosomal Dominant OR X-linked Disorder	Proband with other variant type with some evidence of gene impact	1.5	0.5	0.5	28491681
				Proband with predicted or proven null variant	0	0	0	
				Variant is *de novo*	10.5	10.5	12	25460178;28491681
			Autosomal Recessive Disorder	Two variants (not predicted/proven null) with some evidence of gene impact in *trans*	0	0	0	
				Two variants in *trans* and at least one *de novo* or a predicted/proven null variant				
		Segregation		Candidate gene sequencing	0	0	0	
				Exome/genome or all genes sequenced in linkage region				
	Case-Control				0	0	0	26969752
	**Genetic Evidence Total**				**12**	**11**	**12**	
Experimental Evidence	Functional			Biochemical functions	1	1	0.5	
				Protein interactions				16556865
				Expression				23388215
	Functional Alteration			Patient cells	0	0	0.5	
				Non-patient cells				
	Models			Non-human model organism	0	0	0	
				Cell-culture model				
	Rescue			Rescue in human				
				Rescue in non-human model organism				
				Rescue in cell culture model				
				Rescue in patient cells				
	**Experimental Evidence Total**				**1**	**1**	**1**	
**Total Points**					**13**	**12**	**13**	
**Summary Classification**					**Definitive**			

CAV3

Evidence Type					Points Counted G1	G2	G3	PMID
Genetic Evidence	Case-Level	Variant	Autosomal Dominant OR X-linked Disorder	Proband with other variant type with some evidence of gene impact	0	0	1	17060380;26132555;24917393;24021552
				Proband with predicted or proven null variant	0	0	0	
				Variant is *de novo*	3.5	2	3	17060380
			Autosomal Recessive Disorder	Two variants (not predicted/proven null) with some evidence of gene impact in *trans*	0	0	0	
				Two variants in *trans* and at least one *de novo* or a predicted/proven null variant				
		Segregation		Candidate gene sequencing	0	0	0	
				Exome/genome or all genes sequenced in linkage region				
	Case-Control				0	0	0	
	**Genetic Evidence Total**				**3.5**	**2**	**4**	
Experimental Evidence	Functional			Biochemical functions	1	1.5	0	
				Protein interactions				17060380;24021552;23640888
				Expression				14663034
	Functional Alteration			Patient cells	0.5	1.5	0.5	
				Non-patient cells				17060380;17275750;23640888;23541953
	Models			Non-human model organism	0	0	0	
				Cell-culture model				
	Rescue			Rescue in human				
				Rescue in non-human model organism				
				Rescue in cell culture model				
				Rescue in patient cells				
	**Experimental Evidence Total**				**1.5**	**3**	**0.5**	
**Total Points**					**5**	**5**	**4.5**	
**Summary Classification**					**Limited**			

KCNE1

Evidence Type					Points Counted G1	Points Counted G2	Points Counted G3	PMID
Genetic Evidence	Case-Level	Variant	Autosomal Dominant OR X-linked Disorder	Proband with other variant type with some evidence of gene impact	1.5	2.85	0.75	21712262;17341399;9445165;14499862;9354802;10400998;29672598;16155735
				Proband with predicted or proven null variant	0	0	0	
				Variant is *de novo*	0	0	0	
			Autosomal Recessive Disorder	Two variants (not predicted/proven null) with some evidence of gene impact in *trans*	0	0	0	
				Two variants in *trans* and at least one *de novo* or a predicted/proven null variant				
		Segregation		Candidate gene sequencing	0	0	0	9354783;9445165;29625280;9354802
				Exome/genome or all genes sequenced in linkage region				
	Case-Control				0	0	0	
	Genetic Evidence Total				**1.5**	**2.85**	**0.75**	
Experimental Evidence	Functional			Biochemical functions	0.5	0	1	1939241
				Protein interactions				9201970;20196769
				Expression				
	Functional Alteration			Patient cells	1.5	2	2	
				Non-patient cells				9354802;10400998;29625280;16945797;14499862;17341399;19907016;27076034;14499862
	Models			Non-human model organism	2	1	2	27076034;11320260
				Cell-culture model				
	Rescue			Rescue in human				
				Rescue in non-human model organism				
				Rescue in cell culture model				
				Rescue in patient cells				
	Experimental Evidence Total				**4**	**3**	**5**	
**Total Points**					**5.5**	**5.85**	**5.75**	
**Summary Classification**					**Limited**			

KCNE2

Evidence Type					Points Counted G1	Points Counted G2	Points Counted G3	PMID
Genetic Evidence	Case-Level	Variant	Autosomal Dominant OR X-linked Disorder	Proband with other variant type with some evidence of gene impact	0	1.5	1.6	10219239;28794082;18006462;12185453;23631727
				Proband with predicted or proven null variant	0	0	0	
				Variant is *de novo*	0	0	0	
			Autosomal Recessive Disorder	Two variants (not predicted/proven null) with some evidence of gene impact in *trans*	0	0	0	
				Two variants in *trans* and at least one *de novo* or a predicted/proven null variant				
		Segregation		Candidate gene sequencing	0	0	0	
				Exome/genome or all genes sequenced in linkage region				
	Case-Control				0	1	0	10219239;12185453;28794082;23714088
	Genetic Evidence Total				**0**	**2.5**	**1.6**	
Experimental Evidence	Functional			Biochemical functions	1.5	0.5	0	10219239;15292247;11927665
				Protein interactions				11101505;12185453
				Expression				
	Functional Alteration			Patient cells	0.5	2	0.5	
				Non-patient cells				10219239;18006462;12185453;12923204;
	Models			Non-human model organism	0	0	0	
				Cell-culture model				
	Rescue			Rescue in human				
				Rescue in non-human model organism				
				Rescue in cell culture model				
				Rescue in patient cells				
	Experimental Evidence Total				**2**	**2.5**	**0.5**	
**Total Points**					**2**	**5**	**2.1**	
**Summary Classification**					**Disputed**			

KCNH2

Evidence Type					Points Counted			PMID
					**G1**	**G2**	**G3**	
Genetic Evidence	Case-Level	Variant	**Autosomal Dominant OR X-linked Disorder**	Proband with other variant type with some evidence of gene impact	0.5	4.5	0	19668779;19187913;8635257;7889573;21240260;19070294;21109023
				Proband with predicted or proven null variant	9	1.5	9	15840476;10973849;7889573;18551196;
				Variant is *de novo*	0	0	2	7889573
			**Autosomal Recessive Disorder**	Two variants (not predicted/proven null) with some evidence of gene impact in *trans*	0	0	0	
				Two variants in *trans* and at least one *de novo* or a predicted/proven null variant				
		**Segregation**		Candidate gene sequencing	1.5	3	3	7889573;19668779;19187913;8635257;
				Exome/genome or all genes sequenced in linkage region				
	**Case-Control**				1	3	0	24606995;19841300
	**Genetic Evidence Total**				**12**	**12**	**12**	
Experimental Evidence	**Functional**			Biochemical functions	2	1.5	0.5	7736582;20850565
				Protein interactions				18551196
				Expression				7889573;9012748
	**Functional Alteration**			Patient cells	1.5	2	2	21240260;28433559;
				Non-patient cells				16432067;23997099;21536673;19187913;10753933
	**Models**			Non-human model organism	4	2.5	4	12612061
				Cell-culture model				15760896
	**Rescue**			Rescue in human				
				Rescue in non-human model organism				21458413;23303164
				Rescue in cell culture model				21536673
				Rescue in patient cells				24213244
	**Experimental Evidence Total**				**6**	**6**	**6**	
**Total Points**					**18**	**18**	**18**	
**Summary Classification**					**Definitive**			

KCNJ2

Evidence Type					Points Counted			PMID
					**G1**	**G2**	**G3**	
Genetic Evidence	Case-Level	Variant	**Autosomal Dominant OR X-linked Disorder**	Proband with other variant type with some evidence of gene impact	0	1.5	2.6	20111058;24395924;23644778;22589293;15276028;28003625
				Proband with predicted or proven null variant	0	0	0	21148745;
				Variant is *de novo*	0	0	0	11371347;
			**Autosomal Recessive Disorder**	Two variants (not predicted/proven null) with some evidence of gene impact in *trans*	0	0	0	
				Two variants in *trans* and at least one *de novo* or a predicted/proven null variant				
		**Segregation**		Candidate gene sequencing	0	0	0	11371347;16571646;29017447;12148092;15276028
				Exome/genome or all genes sequenced in linkage region				
	**Case-Control**				0	0	0	
	**Genetic Evidence Total**				**0**	**1.5**	**2.6**	
Experimental Evidence	**Functional**			Biochemical functions	0.5	0	0	11371347
				Protein interactions				
				Expression				
	**Functional Alteration**			Patient cells	1.5	0.5	1.5	
				Non-patient cells				15831539;16541386;17582433;23644778;22589293;15276028
	**Models**			Non-human model organism	1	0	0	
				Cell-culture model				
	**Rescue**			Rescue in human				
				Rescue in non-human model organism				
				Rescue in cell culture model				21493816
				Rescue in patient cells				
	**Experimental Evidence Total**				**3**	**0.5**	**1.5**	
**Total Points**					**3**	**2**	**4.1**	
**Summary Classification**					**Limited**			

KCNJ5

Evidence Type				G1	G2	G3	PMID
				**Points Counted**			
Genetic Evidence	Case-Level – Variant	Autosomal Dominant OR X-linked Disorder	Proband with other variant type with some evidence of gene impact	0	0	0.5	20560207
			Proband with predicted or proven null variant	0	0	0	
			Variant is *de novo*	0	0	0	
		Autosomal Recessive Disorder	Two variants (not predicted/proven null) with some evidence of gene impact in *trans*	0	0	0	
			Two variants in *trans* and at least one *de novo* or a predicted/proven null variant				
	Case-Level	Segregation	Candidate gene sequencing	0	0	0	23872692;25417227
			Exome/genome or all genes sequenced in linkage region				
	Case-Control			0	0	0	
	**Genetic Evidence Total**			**0**	**0**	**0**	
Experimental Evidence	Functional		Biochemical functions	0	1	1	24148898
			Protein interactions				
			Expression				24148898;20560207
	Functional Alteration		Patient cells	0	0.5	0.5	
			Non-patient cells				24574546;20560207
	Models		Non-human model organism	0	0	0	
			Cell-culture model				
	Rescue		Rescue in human				
			Rescue in non-human model organism				
			Rescue in cell culture model				
			Rescue in patient cells				
	**Experimental Evidence Total**			**0**	**1.5**	**1.5**	
**Total Points**				**0**	**1.5**	**2**	
**Summary Classification**				**Disputed**			

KCNQ1

Evidence Type				G1	G2	G3	PMID
				**Points Counted**			
Genetic Evidence	Case-Level – Variant	Autosomal Dominant OR X-linked Disorder	Proband with other variant type with some evidence of gene impact	1	3.5	2.5	17655673;15950200;20660394;8528244;25889101;10220144;
			Proband with predicted or proven null variant	1.5	1.5	7.5	22739119;8528244
			Variant is *de novo*	3.5	2	0	8872472;10220144;25139741
		Autosomal Recessive Disorder	Two variants (not predicted/proven null) with some evidence of gene impact in *trans*	0		0	
			Two variants in *trans* and at least one *de novo* or a predicted/proven null variant				
	Case-Level	Segregation	Candidate gene sequencing	1.5	2.5	3	8528244;10220144;15950200;25139741;20660394;25889101
			Exome/genome or all genes sequenced in linkage region				
	Case-Control			6	3	0	19841300;11216980
	**Genetic Evidence Total**			**12**	**12**	**12**	
Experimental Evidence	Functional		Biochemical functions	1	0.5	0	8900283;9108097
			Protein interactions				
			Expression				25889101
	Functional Alteration		Patient cells	1.5	2	2	20660394;22739119;25889101
			Non-patient cells				9323054;9312006;20421371;17655673;15950200;25139741;20660394;22739119;16246960;15358555;22095730
	Models		Non-human model organism	4	5	4	15498462;18464931;17360457
			Cell-culture model				
	Rescue		Rescue in human				
			Rescue in non-human model organism				
			Rescue in cell culture model				
			Rescue in patient cells				25889101
	**Experimental Evidence Total**			**6**	**6**	**6**	
**Total Points**				**18**	**18**	**18**	
**Summary Classification**				**Definitive**			

SCN4B

Evidence Type					Points Counted G1	G2	G3	PMID
Genetic Evidence	Case-Level	Variant	Autosomal Dominant OR X-linked Disorder	Proband with other variant type with some evidence of gene impact	0.5	0.75	0.5	17592081;20226894;23861362;23631430
				Proband with predicted or proven null variant	0	0	0	
				Variant is *de novo*	0	0	0	
			Autosomal Recessive Disorder	Two variants (not predicted/proven null) with some evidence of gene impact in *trans*	0	0	0	
				Two variants in *trans* and at least one *de novo* or a predicted/proven null variant				
		Segregation		Candidate gene sequencing	0	0	0	17592081
				Exome/genome or all genes sequenced in linkage region				
	**Case-Control**				0	0	0	
	**Genetic Evidence Total**				**0.5**	**0.75**	**0.5**	
Experimental Evidence	Functional			Biochemical functions	1	0.5	0	
				Protein interactions				15007009;17592081
				Expression				12930796
	Functional Alteration			Patient cells	0.5	1.5	0.5	
				Non-patient cells				17592081;20226894
	Models			Non-human model organism	0	0	0	
				Cell-culture model				
	Rescue			Rescue in human				
				Rescue in non-human model organism				
				Rescue in cell culture model				
				Rescue in patient cells				
	**Experimental Evidence Total**				**1.5**	**2**	**0.5**	
**Total Points**					**2**	**2.75**	**1**	
**Summary Classification**					**Disputed**			

SCN5A

Evidence Type					Points Counted G1	G2	G3	PMID
Genetic Evidence	Case-Level	Variant	Autosomal Dominant OR X-linked Disorder	Proband with other variant type with some evidence of gene impact	3.5	2	1	10377081;24871449;27566755;21076026;26467377;14654377;23963187;12673799;12650885
				Proband with predicted or proven null variant	0	0	0	
				Variant is *de novo*	6	6	8	11535573;12123767;15485686;22721569;10200053;19808432;
			Autosomal Recessive Disorder	Two variants (not predicted/proven null) with some evidence of gene impact in *trans*	0	0	0	
				Two variants in *trans* and at least one *de novo* or a predicted/proven null variant				
		Segregation		Candidate gene sequencing	1.5	1	3	7889574;28782696;10627139;10590249
				Exome/genome or all genes sequenced in linkage region				
	**Case-Control**				1	3	1	19841300;10508990
	**Genetic Evidence Total**				**12**	**12**	**12**	
Experimental Evidence	Functional			Biochemical functions	1	0	0	1309946;
				Protein interactions				
				Expression				1309946;
	Functional Alteration			Patient cells	1.5	2	2	23998552;
				Non-patient cells				7651517;17698727;21799153;21076026;14654377;10807877;8917568;9495298;
	Models			Non-human model organism	4	4	4	
				Cell-culture model				
	Rescue			Rescue in human				
				Rescue in non-human model organism				11533705;14736542;17145985
				Rescue in cell culture model				
				Rescue in patient cells				
	**Experimental Evidence Total**				**6**	**6**	**6**	
**Total Points**					**18**	**18**	**18**	
**Summary Classification**					**Definitive**			

SNTA1

Summary Classification					Points Counted G1	G2	G3	PMID
Genetic Evidence	Case-Level	Variant	Autosomal Dominant OR X-linked Disorder	Proband with other variant type with some evidence of gene impact	0.5	0.5	0.75	19684871;18591664;27028743
				Proband with predicted or proven null variant	0	0	0	
				Variant is *de novo*	0	0	1	19684871
			Autosomal Recessive Disorder	Two variants (not predicted/proven null) with some evidence of gene impact in *trans*	0	0	0	
				Two variants in *trans* and at least one *de novo* or a predicted/proven null variant				
		Segregation		Candidate gene sequencing	0	0	0	19684871
				Exome/genome or all genes sequenced in linkage region				
	Case-Control				0	0	0	
	**Genetic Evidence Total**				**0.5**	**0.5**	**1.75**	
Experimental Evidence	Functional			Biochemical functions	0.5	1.5	0.5	18591664
				Protein interactions				18591664;19684871
				Expression				
	Functional Alteration			Patient cells	2	2	1	
				Non-patient cells				19684871;18591664;27028743;23376825
	Models			Non-human model organism	0	0	0	
				Cell-culture model				
	Rescue			Rescue in human				
				Rescue in non-human model organism				
				Rescue in cell culture model				
				Rescue in patient cells				
	**Experimental Evidence Total**				**2.5**	**3.5**	**1.5**	
**Total Points**					**3**	**4**	**3.25**	
**Summary Classification**					**Disputed**			

TRDN

Evidence Type					Points Counted G1	G2	G3	PMID
Genetic Evidence	Case-Level	Variant	Autosomal Dominant OR X-linked Disorder	Proband with other variant type with some evidence of gene impact	0	0	0	25922419;26768964
				Proband with predicted or proven null variant	0	0	0	
				Variant is *de novo*	0	0	0	
			Autosomal Recessive Disorder	Two variants (not predicted/proven null) with some evidence of gene impact in *trans*	11	11.5	3	
				Two variants in *trans* and at least one *de novo* or a predicted/proven null variant				
		Segregation		Candidate gene sequencing	0	0	0	26768964
				Exome/genome or all genes sequenced in linkage region				
	Case-Control				0	0	0	
	**Genetic Evidence Total**				**11**	**11.5**	**3**	
Experimental Evidence	Functional			Biochemical functions	0	0	0	
				Protein interactions				
				Expression				
	Functional Alteration			Patient cells	0	0	0	
				Non-patient cells				
	Models			Non-human model organism	0	0	0	
				Cell-culture model				
	Rescue			Rescue in human				
				Rescue in non-human model organism				
				Rescue in cell culture model				
				Rescue in patient cells				
	**Experimental Evidence Total**				**0**	**0**	**0**	
**Total Points**					**11**	**11.5**	**3**	
**Summary Classification**					**Strong**			

Supplemental references

AKAP9

1. Marx SO, Kurokawa J, Reiken S, et al. Requirement of a macromolecular signaling complex for beta adrenergic receptor modulation of the KCNQ1-KCNE1 potassium channel. Science 2002;295:496-469.
2. Chen L, Marquardt ML, Tester DJ, Sampson KJ, Ackerman MJ, Kass RS. Mutation of an A-kinase-anchoring protein causes long-QT syndrome. Proc Natl Acad Sci U S A 2007;104:20990-20995.
3. Li Y, Chen L, Kass RS, Dessauer CW. The A-kinase anchoring protein Yotiao facilitates complex formation between adenylyl cyclase type 9 and the IKs potassium channel in heart. J Biol Chem 2012;287:29815-29824.
4. Dvir M, Strulovich R, Sachyani D, et al. Long QT mutations at the interface between KCNQ1 helix C and KCNE1 disrupt I(KS) regulation by PKA and PIP(2). J Cell Sci 2014;127:3943-3955.
5. Shigemizu D, Aiba T, Nakagawa H, et al. Exome Analyses of Long QT Syndrome Reveal Candidate Pathogenic Mutations in Calmodulin-Interacting Genes. PLoS One 2015;10:e0130329.
6. Jimenez-Jaimez J, Peinado R, Grima EZ, et al. Diagnostic Approach to Unexplained Cardiac Arrest (from the FIVI-Gen Study). Am J Cardiol 2015;116:894-899.
7. Maltese PE, Orlova N, Krasikova E, et al. Gene-Targeted Analysis of Clinically Diagnosed Long QT Russian Families. Int Heart J 2017;58:81-87.
8. Neubauer J, Lecca MR, Russo G, et al. Exome analysis in 34 sudden unexplained death (SUD) victims mainly identified variants in channelopathy-associated genes. Int J Legal Med 2018;132:1057-1065.

ANK2

9. Tuvia S, Buhusi M, Davis L, Reedy M, Bennett V. Ankyrin-B is required for intracellular sorting of structurally diverse Ca2+ homeostasis proteins. J Cell Biol 1999;147:995-1008.
10. Mohler PJ, Schott JJ, Gramolini AO, et al. Ankyrin-B mutation causes type 4 long-QT cardiac arrhythmia and sudden cardiac death. Nature 2003;421:634-639.
11. Mohler PJ, Splawski I, Napolitano C, et al. A cardiac arrhythmia syndrome caused by loss of ankyrin-B function. Proc Natl Acad Sci U S A 2004;101:9137-9142.
12. Sherman J, Tester DJ, Ackerman MJ. Targeted mutational analysis of ankyrin-B in 541 consecutive, unrelated patients referred for long QT syndrome genetic testing and 200 healthy subjects. Heart Rhythm 2005;2:1218-1223.
13. Zhou X, Shimizu M, Konno T, et al. Analysis of ankyrin-B gene mutations in patients with long QT syndrome. Nan Fang Yi Ke Da Xue Xue Bao 2006;26:901-903.

14. Mohler PJ, Le Scouarnec S, Denjoy I, et al. Defining the cellular phenotype of "ankyrin-B syndrome" variants: human ANK2 variants associated with clinical phenotypes display a spectrum of activities in cardiomyocytes. Circulation 2007;115:432-441.
15. Shigemizu D, Aiba T, Nakagawa H, et al. Exome Analyses of Long QT Syndrome Reveal Candidate Pathogenic Mutations in Calmodulin-Interacting Genes. PLoS One 2015;10:e0130329.
16. Ichikawa M, Aiba T, Ohno S, et al. Phenotypic Variability of ANK2 Mutations in Patients With Inherited Primary Arrhythmia Syndromes. Circ J 2016;80:2435-2442.
17. Swayne LA, Murphy NP, Asuri S, et al. Novel Variant in the ANK2 Membrane-Binding Domain Is Associated With Ankyrin-B Syndrome and Structural Heart Disease in a First Nations Population With a High Rate of Long QT Syndrome. Circ Cardiovasc Genet 2017;10: e001537.
18. Maltese PE, Orlova N, Krasikova E, et al. Gene-Targeted Analysis of Clinically Diagnosed Long QT Russian Families. Int Heart J 2017;58:81-87.

CACNA1C

19. Splawski I, Timothy KW, Decher N, et al. Severe arrhythmia disorder caused by cardiac L-type calcium channel mutations. Proc Natl Acad Sci U S A 2005;102:8089-8096.
20. Boczek NJ, Best JM, Tester DJ, et al. Exome sequencing and systems biology converge to identify novel mutations in the L-type calcium channel, CACNA1C, linked to autosomal dominant long QT syndrome. Circ Cardiovasc Genet 2013;6:279-289.
21. Fukuyama M, Wang Q, Kato K, et al. Long QT syndrome type 8: novel CACNA1C mutations causing QT prolongation and variant phenotypes. Europace 2014;16:1828-1837.
22. Wemhoner K, Friedrich C, Stallmeyer B, et al. Gain-of-function mutations in the calcium channel CACNA1C (Cav1.2) cause non-syndromic long-QT but not Timothy syndrome. J Mol Cell Cardiol 2015;80:186-195.
23. Landstrom AP, Boczek NJ, Ye D, et al. Novel long QT syndrome-associated missense mutation, L762F, in CACNA1C-encoded L-type calcium channel imparts a slower inactivation tau and increased sustained and window current. Int J Cardiol 2016;220:290-298.

CALM1

24. Shamgar L, Ma L, Schmitt N, et al. Calmodulin is essential for cardiac IKS channel gating and assembly: impaired function in long-QT mutations. Circ Res 2006;98:1055-1063.
25. Crotti L, Johnson CN, Graf E, et al. Calmodulin mutations associated with recurrent cardiac arrest in infants. Circulation 2013;127:1009-1017.

26. Marsman RF, Barc J, Beekman L, et al. A mutation in CALM1 encoding calmodulin in familial idiopathic ventricular fibrillation in childhood and adolescence. J Am Coll Cardiol 2014;63:259-266.
27. Limpitikul WB, Dick IE, Joshi-Mukherjee R, Overgaard MT, George AL, Jr., Yue DT. Calmodulin mutations associated with long QT syndrome prevent inactivation of cardiac L-type Ca(2+) currents and promote proarrhythmic behavior in ventricular myocytes. J Mol Cell Cardiol 2014;74:115-124.
28. Sondergaard MT, Tian X, Liu Y, et al. Arrhythmogenic Calmodulin Mutations Affect the Activation and Termination of Cardiac Ryanodine Receptor-mediated Ca2+ Release. J Biol Chem 2015;290:26151-26162.
29. Boczek NJ, Gomez-Hurtado N, Ye D, et al. Spectrum and Prevalence of CALM1-, CALM2-, and CALM3-Encoded Calmodulin Variants in Long QT Syndrome and Functional Characterization of a Novel Long QT Syndrome-Associated Calmodulin Missense Variant, E141G. Circ Cardiovasc Genet 2016;9:136-146.
30. Pipilas DC, Johnson CN, Webster G, et al. Novel calmodulin mutations associated with congenital long QT syndrome affect calcium current in human cardiomyocytes. Heart Rhythm 2016;13:2012-2019.
31. Rocchetti M, Sala L, Dreizehnter L, et al. Elucidating arrhythmogenic mechanisms of long-QT syndrome CALM1-F142L mutation in patient-specific induced pluripotent stem cell-derived cardiomyocytes. Cardiovasc Res 2017;113:531-541.
32. Crotti L, Spazzolini C, Tester DJ, Ghidoni A, Baruteau AE, Beckmann BM, Behr ER, Bennett JS, Bezzina CR, Bhuiyan ZA, Celiker A, Cerrone M, Dagradi F, De Ferrari GM, Etheridge SP, Fatah M, Garcia-Pavia P, Al-Ghamdi S, Hamilton RM, Al-Hassnan ZN, Horie M, Jimenez-Jaimez J, Kanter RJ, Kaski JP, Kotta MC, Lahrouchi N, Makita N, Norrish G, Odland HH, Ohno S, Papagiannis J, Parati G, Sekarski N, Tveten K, Vatta M, Webster G, Wilde AAM, Wojciak J, George AL, Ackerman MJ and Schwartz PJ. Calmodulin mutations and life-threatening cardiac arrhythmias: insights from the International Calmodulinopathy Registry. *Eur Heart J*. 2019;40: 2964-2975.

CALM2

33. Shamgar L, Ma L, Schmitt N, et al. Calmodulin is essential for cardiac IKS channel gating and assembly: impaired function in long-QT mutations. Circ Res 2006;98:1055-1063.
34. Crotti L, Johnson CN, Graf E, et al. Calmodulin mutations associated with recurrent cardiac arrest in infants. Circulation 2013;127:1009-1017.
35. Makita N, Yagihara N, Crotti L, et al. Novel calmodulin mutations associated with congenital arrhythmia susceptibility. Circ Cardiovasc Genet 2014;7:466-474.
36. Yin G, Hassan F, Haroun AR, et al. Arrhythmogenic calmodulin mutations disrupt intracellular cardiomyocyte Ca2+ regulation by distinct mechanisms. J Am Heart Assoc 2014;3:e000996.

37. Limpitikul WB, Dick IE, Joshi-Mukherjee R, Overgaard MT, George AL, Jr., Yue DT. Calmodulin mutations associated with long QT syndrome prevent inactivation of cardiac L-type Ca(2+) currents and promote proarrhythmic behavior in ventricular myocytes. J Mol Cell Cardiol 2014;74:115-124.
38. Sondergaard MT, Tian X, Liu Y, et al. Arrhythmogenic Calmodulin Mutations Affect the Activation and Termination of Cardiac Ryanodine Receptor-mediated Ca2+ Release. J Biol Chem 2015;290:26151-26162.
39. Boczek NJ, Gomez-Hurtado N, Ye D, et al. Spectrum and Prevalence of CALM1-, CALM2-, and CALM3-Encoded Calmodulin Variants in Long QT Syndrome and Functional Characterization of a Novel Long QT Syndrome-Associated Calmodulin Missense Variant, E141G. Circ Cardiovasc Genet 2016;9:136-146.
40. Pipilas DC, Johnson CN, Webster G, et al. Novel calmodulin mutations associated with congenital long QT syndrome affect calcium current in human cardiomyocytes. Heart Rhythm 2016;13:2012-2019.
41. Sun J, MacKinnon R. Cryo-EM Structure of a KCNQ1/CaM Complex Reveals Insights into Congenital Long QT Syndrome. Cell 2017;169:1042-1050.
42. Yamamoto Y, Makiyama T, Harita T, et al. Allele-specific ablation rescues electrophysiological abnormalities in a human iPS cell model of long-QT syndrome with a CALM2 mutation. Hum Mol Genet 2017;26:1670-1677.
43. Limpitikul WB, Dick IE, Tester DJ, et al. A Precision Medicine Approach to the Rescue of Function on Malignant Calmodulinopathic Long-QT Syndrome. Circ Res 2017;120:39-48.
44. Crotti L, Spazzolini C, Tester DJ, Ghidoni A, Baruteau AE, Beckmann BM, Behr ER, Bennett JS, Bezzina CR, Bhuiyan ZA, Celiker A, Cerrone M, Dagradi F, De Ferrari GM, Etheridge SP, Fatah M, Garcia-Pavia P, Al-Ghamdi S, Hamilton RM, Al-Hassnan ZN, Horie M, Jimenez-Jaimez J, Kanter RJ, Kaski JP, Kotta MC, Lahrouchi N, Makita N, Norrish G, Odland HH, Ohno S, Papagiannis J, Parati G, Sekarski N, Tveten K, Vatta M, Webster G, Wilde AAM, Wojciak J, George AL, Ackerman MJ and Schwartz PJ. Calmodulin mutations and life-threatening cardiac arrhythmias: insights from the International Calmodulinopathy Registry. *Eur Heart J*. 2019;40: 2964-2975.

CALM3

45. Shamgar L, Ma L, Schmitt N, et al. Calmodulin is essential for cardiac IKS channel gating and assembly: impaired function in long-QT mutations. Circ Res 2006;98:1055-1063.
46. Crotti L, Johnson CN, Graf E, et al. Calmodulin mutations associated with recurrent cardiac arrest in infants. Circulation 2013;127:1009-1017.
47. Reed GJ, Boczek NJ, Etheridge SP, Ackerman MJ. CALM3 mutation associated with long QT syndrome. Heart Rhythm 2015;12:419-422.
48. Chaix MA, Koopmann TT, Goyette P, et al. Novel CALM3 mutations in pediatric long QT syndrome patients support a CALM3-specific calmodulinopathy. HeartRhythm Case Rep 2016;2:250-254.

49. Boczek NJ, Gomez-Hurtado N, Ye D, et al. Spectrum and Prevalence of CALM1-, CALM2-, and CALM3-Encoded Calmodulin Variants in Long QT Syndrome and Functional Characterization of a Novel Long QT Syndrome-Associated Calmodulin Missense Variant, E141G. Circ Cardiovasc Genet 2016;9:136-146.
50. Crotti L, Spazzolini C, Tester DJ, Ghidoni A, Baruteau AE, Beckmann BM, Behr ER, Bennett JS, Bezzina CR, Bhuiyan ZA, Celiker A, Cerrone M, Dagradi F, De Ferrari GM, Etheridge SP, Fatah M, Garcia-Pavia P, Al-Ghamdi S, Hamilton RM, Al-Hassnan ZN, Horie M, Jimenez-Jaimez J, Kanter RJ, Kaski JP, Kotta MC, Lahrouchi N, Makita N, Norrish G, Odland HH, Ohno S, Papagiannis J, Parati G, Sekarski N, Tveten K, Vatta M, Webster G, Wilde AAM, Wojciak J, George AL, Ackerman MJ and Schwartz PJ. Calmodulin mutations and life-threatening cardiac arrhythmias: insights from the International Calmodulinopathy Registry. *Eur Heart J.* 2019;40: 2964-2975.

CAV3

51. Cagliani R, Bresolin N, Prelle A, et al. A CAV3 microdeletion differentially affects skeletal muscle and myocardium. Neurology 2003;61:1513-1519.
52. Vatta M, Ackerman MJ, Ye B, et al. Mutant caveolin-3 induces persistent late sodium current and is associated with long-QT syndrome. Circulation 2006;114:2104-2112.
53. Cronk LB, Ye B, Kaku T, et al. Novel mechanism for sudden infant death syndrome: persistent late sodium current secondary to mutations in caveolin-3. Heart Rhythm 2007;4:161-166.
54. Hedley PL, Kanters JK, Dembic M, et al. The role of CAV3 in long-QT syndrome: clinical and functional assessment of a caveolin-3/Kv11.1 double heterozygote versus caveolin-3 single heterozygote. Circ Cardiovasc Genet 2013;6:452-461.
55. Vaidyanathan R, Vega AL, Song C, et al. The interaction of caveolin 3 protein with the potassium inward rectifier channel Kir2.1: physiology and pathology related to long qt syndrome 9 (LQT9). J Biol Chem 2013;288:17472-17480.
56. Cheng J, Valdivia CR, Vaidyanathan R, Balijepalli RC, Ackerman MJ, Makielski JC. Caveolin-3 suppresses late sodium current by inhibiting nNOS-dependent S-nitrosylation of SCN5A. J Mol Cell Cardiol 2013;61:102-110.
57. Lariccia V, Nasti AA, Alessandrini F, et al. Identification and functional analysis of a new putative caveolin-3 variant found in a patient with sudden unexplained death. J Biomed Sci 2014;21:58.
58. Shigemizu D, Aiba T, Nakagawa H, et al. Exome Analyses of Long QT Syndrome Reveal Candidate Pathogenic Mutations in Calmodulin-Interacting Genes. PLoS One 2015;10:e0130329.

KCNE1

59. Takumi T, Moriyoshi K, Aramori I, et al. Alteration of channel activities and gating by mutations of slow ISK potassium channel. J Biol Chem 1991;266:22192-22198.

60. Splawski I, Tristani-Firouzi M, Lehmann MH, Sanguinetti MC, Keating MT. Mutations in the hminK gene cause long QT syndrome and suppress IKs function. Nat Genet 1997;17:338-340.
61. Schulze-Bahr E, Wang Q, Wedekind H, et al. KCNE1 mutations cause jervell and Lange-Nielsen syndrome. Nat Genet 1997;17:267-268.
62. Romey G, Attali B, Chouabe C, et al. Molecular mechanism and functional significance of the MinK control of the KvLQT1 channel activity. J Biol Chem 1997;272:16713-16716.
63. Duggal P, Vesely MR, Wattanasirichaigoon D, Villafane J, Kaushik V, Beggs AH. Mutation of the gene for IsK associated with both Jervell and Lange-Nielsen and Romano-Ward forms of Long-QT syndrome. Circulation 1998;97:142-146.
64. Bianchi L, Shen Z, Dennis AT, et al. Cellular dysfunction of LQT5-minK mutants: abnormalities of IKs, IKr and trafficking in long QT syndrome. Hum Mol Genet 1999;8:1499-1507.
65. Hoppe UC, Marban E, Johns DC. Distinct gene-specific mechanisms of arrhythmia revealed by cardiac gene transfer of two long QT disease genes, HERG and KCNE1. Proc Natl Acad Sci U S A 2001;98:5335-5340.
66. Ma L, Lin C, Teng S, et al. Characterization of a novel Long QT syndrome mutation G52R-KCNE1 in a Chinese family. Cardiovasc Res 2003;59:612-619.
67. Lai LP, Su YN, Hsieh FJ, et al. Denaturing high-performance liquid chromatography screening of the long QT syndrome-related cardiac sodium and potassium channel genes and identification of novel mutations and single nucleotide polymorphisms. J Hum Genet 2005;50:490-496.
68. Wu DM, Lai LP, Zhang M, et al. Characterization of an LQT5-related mutation in KCNE1, Y81C: implications for a role of KCNE1 cytoplasmic domain in IKs channel function. Heart Rhythm 2006;3:1031-1040.
69. Ohno S, Zankov DP, Yoshida H, et al. N- and C-terminal KCNE1 mutations cause distinct phenotypes of long QT syndrome. Heart Rhythm 2007;4:332-340.
70. Harmer SC, Wilson AJ, Aldridge R, Tinker A. Mechanisms of disease pathogenesis in long QT syndrome type 5. Am J Physiol Cell Physiol 2010;298:C263-273.
71. Zheng R, Thompson K, Obeng-Gyimah E, et al. Analysis of the interactions between the C-terminal cytoplasmic domains of KCNQ1 and KCNE1 channel subunits. Biochem J 2010;428:75-84.
72. Nof E, Barajas-Martinez H, Eldar M, et al. LQT5 masquerading as LQT2: a dominant negative effect of KCNE1-D85N rare polymorphism on KCNH2 current. Europace 2011;13:1478-1483.
73. Major P, Baczko I, Hiripi L, et al. A novel transgenic rabbit model with reduced repolarization reserve: long QT syndrome caused by a dominant-negative mutation of the KCNE1 gene. Br J Pharmacol 2016;173:2046-2061.
74. Marcondes L, Crawford J, Earle N, et al. Long QT molecular autopsy in sudden unexplained death in the young (1-40 years old): Lessons learnt from an eight year experience in New Zealand. PLoS One 2018;13:e0196078.
75. Lane CM, Giudicessi JR, Ye D, et al. Long QT syndrome type 5-Lite: Defining the clinical phenotype associated with the potentially proarrhythmic p.Asp85Asn-KCNE1 common genetic variant. Heart Rhythm 2018;15:1223-1230.

KCNE2

76. Abbott GW, Sesti F, Splawski I, et al. MiRP1 forms IKr potassium channels with HERG and is associated with cardiac arrhythmia. Cell 1999;97:175-187.
77. Tinel N, Diochot S, Borsotto M, Lazdunski M, Barhanin J. KCNE2 confers background current characteristics to the cardiac KCNQ1 potassium channel. EMBO J 2000;19:6326-6330.
78. Isbrandt D, Friederich P, Solth A, et al. Identification and functional characterization of a novel KCNE2 (MiRP1) mutation that alters HERG channel kinetics. J Mol Med (Berl) 2002;80:524-532.
79. Weerapura M, Nattel S, Chartier D, Caballero R, Hebert TE. A comparison of currents carried by HERG, with and without coexpression of MiRP1, and the native rapid delayed rectifier current. Is MiRP1 the missing link? J Physiol 2002;540:15-27.
80. Lu Y, Mahaut-Smith MP, Huang CL, Vandenberg JI. Mutant MiRP1 subunits modulate HERG K+ channel gating: a mechanism for pro-arrhythmia in long QT syndrome type 6. J Physiol 2003;551:253-262.
81. Qu J, Kryukova Y, Potapova IA, et al. MiRP1 modulates HCN2 channel expression and gating in cardiac myocytes. J Biol Chem 2004;279:43497-43502.
82. Gordon E, Panaghie G, Deng L, et al. A KCNE2 mutation in a patient with cardiac arrhythmia induced by auditory stimuli and serum electrolyte imbalance. Cardiovasc Res 2008;77:98-106.
83. Nawathe PA, Kryukova Y, Oren RV, et al. An LQTS6 MiRP1 mutation suppresses pacemaker current and is associated with sinus bradycardia. J Cardiovasc Electrophysiol 2013;24:1021-1027.
84. Aziz PF, Wieand TS, Ganley J, Henderson J, McBride M, Shah MJ. Do LQTS gene single nucleotide polymorphisms alter QTc intervals at rest and during exercise stress testing? Ann Noninvasive Electrocardiol 2013;18:288-293.
85. Roberts JD, Krahn AD, Ackerman MJ, et al. Loss-of-Function KCNE2 Variants: True Monogenic Culprits of Long-QT Syndrome or Proarrhythmic Variants Requiring Secondary Provocation? Circ Arrhythm Electrophysiol 2017;10:e005282.

KCNH2

86. Sanguinetti MC, Jiang C, Curran ME, Keating MT. A mechanistic link between an inherited and an acquired cardiac arrhythmia: HERG encodes the IKr potassium channel. Cell 1995;81:299-307.
87. Wymore RS, Gintant GA, Wymore RT, Dixon JE, McKinnon D, Cohen IS. Tissue and species distribution of mRNA for the IKr-like K+ channel, erg. Circ Res 1997;80:261-268.
88. Kagan A, Yu Z, Fishman GI, McDonald TV. The dominant negative LQT2 mutation A561V reduces wild-type HERG expression. J Biol Chem 2000;275:11241-11248.

89. Tenenbaum M, Lavi S, Magal N, et al. Identification of the gene causing long QT syndrome in an Israeli family. Isr Med Assoc J 2008;10:809-811.
90. Splawski I, Shen J, Timothy KW, et al. Spectrum of mutations in long-QT syndrome genes. KVLQT1, HERG, SCN5A, KCNE1, and KCNE2. Circulation 2000;102:1178-1185.
91. Lees-Miller JP, Guo J, Somers JR, et al. Selective knockout of mouse ERG1 B potassium channel eliminates I(Kr) in adult ventricular myocytes and elicits episodes of abrupt sinus bradycardia. Mol Cell Biol 2003;23:1856-1862.
92. Tester DJ, Will ML, Haglund CM, Ackerman MJ. Compendium of cardiac channel mutations in 541 consecutive unrelated patients referred for long QT syndrome genetic testing. Heart Rhythm 2005;2:507-517.
93. Gong Q, Keeney DR, Molinari M, Zhou Z. Degradation of trafficking-defective long QT syndrome type II mutant channels by the ubiquitin-proteasome pathway. J Biol Chem 2005;280:19419-19425.
94. Anderson CL, Delisle BP, Anson BD, et al. Most LQT2 mutations reduce Kv11.1 (hERG) current by a class 2 (trafficking-deficient) mechanism. Circulation 2006;113:365-373.
95. Amin AS, Herfst LJ, Delisle BP, et al. Fever-induced QTc prolongation and ventricular arrhythmias in individuals with type 2 congenital long QT syndrome. J Clin Invest 2008;118:2552-2561.
96. Kim JA, Lopes CM, Moss AJ, et al. Trigger-specific risk factors and response to therapy in long QT syndrome type 2. Heart Rhythm 2010;7:1797-1805.
97. Kapa S, Tester DJ, Salisbury BA, et al. Genetic testing for long-QT syndrome: distinguishing pathogenic mutations from benign variants. Circulation 2009;120:1752-1760.
98. Itzhaki I, Maizels L, Huber I, et al. Modelling the long QT syndrome with induced pluripotent stem cells. Nature 2011;471:225-229.
99. Amoros I, Jimenez-Jaimez J, Tercedor L, et al. Functional effects of a missense mutation in HERG associated with type 2 long QT syndrome. Heart Rhythm 2011;8:463-470.
100. Gianulis EC, Trudeau MC. Rescue of aberrant gating by a genetically encoded PAS (Per-Arnt-Sim) domain in several long QT syndrome mutant human ether-a-go-go-related gene potassium channels. J Biol Chem 2011;286:22160-22169.
101. Meder B, Scholz EP, Hassel D, et al. Reconstitution of defective protein trafficking rescues Long-QT syndrome in zebrafish. Biochem Biophys Res Commun 2011;408:218-224.
102. Stump MR, Gong Q, Zhou Z. LQT2 nonsense mutations generate trafficking defective NH2-terminally truncated channels by the reinitiation of translation. Am J Physiol Heart Circ Physiol 2013;305:H1397-404.
103. Jou CJ, Barnett SM, Bian JT, Weng HC, Sheng X, Tristani-Firouzi M. An in vivo cardiac assay to determine the functional consequences of putative long QT syndrome mutations. Circ Res 2013;112:826-830.
104. Bellin M, Casini S, Davis RP, et al. Isogenic human pluripotent stem cell pairs reveal the role of a KCNH2 mutation in long-QT syndrome. EMBO J 2013;32:3161-3175.

105. Christiansen M, Hedley PL, Theilade J, et al. Mutations in Danish patients with long QT syndrome and the identification of a large founder family with p.F29L in KCNH2. BMC Med Genet 2014;15:31.
106. Mura M, Mehta A, Ramachandra CJ, et al. The KCNH2-IVS9-28A/G mutation causes aberrant isoform expression and hERG trafficking defect in cardiomyocytes derived from patients affected by Long QT Syndrome type 2. Int J Cardiol 2017;240:367-371.

KCNJ2

107. Plaster NM, Tawil R, Tristani-Firouzi M, et al. Mutations in Kir2.1 cause the developmental and episodic electrical phenotypes of Andersen's syndrome. Cell 2001;105:511-519.
108. Andelfinger G, Tapper AR, Welch RC, Vanoye CG, George AL, Jr., Benson DW. KCNJ2 mutation results in Andersen syndrome with sex-specific cardiac and skeletal muscle phenotypes. Am J Hum Genet 2002;71:663-668.
109. Fodstad H, Swan H, Auberson M, et al. Loss-of-function mutations of the K(+) channel gene KCNJ2 constitute a rare cause of long QT syndrome. J Mol Cell Cardiol 2004;37:593-602.
110. Bendahhou S, Fournier E, Sternberg D, et al. In vivo and in vitro functional characterization of Andersen's syndrome mutations. J Physiol 2005;565:731-741.
111. Ballester LY, Benson DW, Wong B, et al. Trafficking-competent and trafficking-defective KCNJ2 mutations in Andersen syndrome. Hum Mutat 2006;27:388.
112. Lu CW, Lin JH, Rajawat YS, et al. Functional and clinical characterization of a mutation in KCNJ2 associated with Andersen-Tawil syndrome. J Med Genet 2006;43:653-659.
113. Tani Y, Miura D, Kurokawa J, et al. T75M-KCNJ2 mutation causing Andersen-Tawil syndrome enhances inward rectification by changing Mg2+ sensitivity. J Mol Cell Cardiol 2007;43:187-196.
114. Chan HF, Chen ML, Su JJ, Ko LC, Lin CH, Wu RM. A novel neuropsychiatric phenotype of KCNJ2 mutation in one Taiwanese family with Andersen-Tawil syndrome. J Hum Genet 2010;55:186-188.
115. Doi T, Makiyama T, Morimoto T, et al. A novel KCNJ2 nonsense mutation, S369X, impedes trafficking and causes a limited form of Andersen-Tawil syndrome. Circ Cardiovasc Genet 2011;4:253-260.
116. Barajas-Martinez H, Hu D, Ontiveros G, et al. Biophysical and molecular characterization of a novel de novo KCNJ2 mutation associated with Andersen-Tawil syndrome and catecholaminergic polymorphic ventricular tachycardia mimicry. Circ Cardiovasc Genet 2011;4:51-57.
117. Kimura H, Zhou J, Kawamura M, et al. Phenotype variability in patients carrying KCNJ2 mutations. Circ Cardiovasc Genet 2012;5:344-353.
118. Limberg MM, Zumhagen S, Netter MF, et al. Non dominant-negative KCNJ2 gene mutations leading to Andersen-Tawil syndrome with an isolated cardiac phenotype. Basic Res Cardiol 2013;108:353.

119. Marquis-Nicholson R, Prosser DO, Love JM, et al. Array comparative genomic hybridization identifies a heterozygous deletion of the entire KCNJ2 gene as a cause of sudden cardiac death. Circ Cardiovasc Genet 2014;7:17-22.
120. Maltese PE, Orlova N, Krasikova E, et al. Gene-Targeted Analysis of Clinically Diagnosed Long QT Russian Families. Int Heart J 2017;58:81-87.
121. Scheiper S, Hertel B, Beckmann BM, Kaab S, Thiel G, Kauferstein S. Characterization of a novel KCNJ2 sequence variant detected in Andersen-Tawil syndrome patients. BMC Med Genet 2017;18:113.

KCNJ5

122. Yang Y, Yang Y, Liang B, et al. Identification of a Kir3.4 mutation in congenital long QT syndrome. Am J Hum Genet 2010;86:872-880.
123. Wang F, Liu J, Hong L, et al. The phenotype characteristics of type 13 long QT syndrome with mutation in KCNJ5 (Kir3.4-G387R). Heart Rhythm 2013;10:1500-1506.
124. Bond R, Blaufox A, Goldner B, Patel A. Congenital long QT syndrome: a case report of LQT2 and LQT13 in a neonate. Europace 2014;16:1807.
125. Liang B, Nissen JD, Laursen M, et al. G-protein-coupled inward rectifier potassium current contributes to ventricular repolarization. Cardiovasc Res 2014;101:175-184.
126. Kokunai Y, Nakata T, Furuta M, et al. A Kir3.4 mutation causes Andersen-Tawil syndrome by an inhibitory effect on Kir2.1. Neurology 2014;82:1058-1064.

KCNQ1

127. Wang Q, Curran ME, Splawski I, et al. Positional cloning of a novel potassium channel gene: KVLQT1 mutations cause cardiac arrhythmias. Nat Genet 1996;12:17-23.
128. Russell MW, Dick M, 2nd, Collins FS, Brody LC. KVLQT1 mutations in three families with familial or sporadic long QT syndrome. Hum Mol Genet 1996;5:1319-1324.
129. Sanguinetti MC, Curran ME, Zou A, et al. Coassembly of K(V)LQT1 and minK (IsK) proteins to form cardiac I(Ks) potassium channel. Nature 1996;384:80-83.
130. Yang WP, Levesque PC, Little WA, Conder ML, Shalaby FY, Blanar MA. KvLQT1, a voltage-gated potassium channel responsible for human cardiac arrhythmias. Proc Natl Acad Sci U S A 1997;94:4017-4021.
131. Shalaby FY, Levesque PC, Yang WP, et al. Dominant-negative KvLQT1 mutations underlie the LQT1 form of long QT syndrome. Circulation 1997;96:1733-1736.

132. Chouabe C, Neyroud N, Guicheney P, Lazdunski M, Romey G, Barhanin J. Properties of KvLQT1 K+ channel mutations in Romano-Ward and Jervell and Lange-Nielsen inherited cardiac arrhythmias. EMBO J 1997;16:5472-5479.
133. Jongbloed RJ, Wilde AA, Geelen JL, et al. Novel KCNQ1 and HERG missense mutations in Dutch long-QT families. Hum Mutat 1999;13:301-310.
134. Piippo K, Swan H, Pasternack M, et al. A founder mutation of the potassium channel KCNQ1 in long QT syndrome: implications for estimation of disease prevalence and molecular diagnostics. J Am Coll Cardiol 2001;37:562-568.
135. Aizawa Y, Ueda K, Wu LM, et al. Truncated KCNQ1 mutant, A178fs/105, forms hetero-multimer channel with wild-type causing a dominant-negative suppression due to trafficking defect. FEBS Lett 2004;574:145-150.
136. Casimiro MC, Knollmann BC, Yamoah EN, et al. Targeted point mutagenesis of mouse Kcnq1: phenotypic analysis of mice with point mutations that cause Romano-Ward syndrome in humans. Genomics 2004;84:555-564.
137. Thomas D, Wimmer AB, Karle CA, et al. Dominant-negative I(Ks) suppression by KCNQ1-deltaF339 potassium channels linked to Romano-Ward syndrome. Cardiovasc Res 2005;67:487-497.
138. Brink PA, Crotti L, Corfield V, et al. Phenotypic variability and unusual clinical severity of congenital long-QT syndrome in a founder population. Circulation 2005;112:2602-2610.
139. Aizawa Y, Ueda K, Scornik F, et al. A novel mutation in KCNQ1 associated with a potent dominant negative effect as the basis for the LQT1 form of the long QT syndrome. J Cardiovasc Electrophysiol 2007;18:972-977.
140. Ocorr K, Reeves NL, Wessells RJ, et al. KCNQ potassium channel mutations cause cardiac arrhythmias in Drosophila that mimic the effects of aging. Proc Natl Acad Sci U S A 2007;104:3943-3948.
141. Brunner M, Peng X, Liu GX, et al. Mechanisms of cardiac arrhythmias and sudden death in transgenic rabbits with long QT syndrome. J Clin Invest 2008;118:2246-2259.
142. Kapa S, Tester DJ, Salisbury BA, et al. Genetic testing for long-QT syndrome: distinguishing pathogenic mutations from benign variants. Circulation 2009;120:1752-1760.
143. Moretti A, Bellin M, Welling A, et al. Patient-specific induced pluripotent stem-cell models for long-QT syndrome. N Engl J Med 2010;363:1397-1409.
144. Eldstrom J, Xu H, Werry D, et al. Mechanistic basis for LQT1 caused by S3 mutations in the KCNQ1 subunit of IKs. J Gen Physiol 2010;135:433-448.
145. Egashira T, Yuasa S, Suzuki T, et al. Disease characterization using LQTS-specific induced pluripotent stem cells. Cardiovasc Res 2012;95:419-429.
146. Heijman J, Spatjens RL, Seyen SR, et al. Dominant-negative control of cAMP-dependent IKs upregulation in human long-QT syndrome type 1. Circ Res 2012;110:211-219.
147. Spatjens RL, Bebarova M, Seyen SR, et al. Long-QT mutation p.K557E-Kv7.1: dominant-negative suppression of IKs, but preserved cAMP-dependent up-regulation. Cardiovasc Res 2014;104:216-225.

148. Ma D, Wei H, Lu J, et al. Characterization of a novel KCNQ1 mutation for type 1 long QT syndrome and assessment of the therapeutic potential of a novel IKs activator using patient-specific induced pluripotent stem cell-derived cardiomyocytes. Stem Cell Res Ther 2015;6:39.

SCN4B

149. Yu FH, Westenbroek RE, Silos-Santiago I, et al. Sodium channel beta4, a new disulfide-linked auxiliary subunit with similarity to beta2. J Neurosci 2003;23:7577-7585.
150. Maier SK, Westenbroek RE, McCormick KA, Curtis R, Scheuer T, Catterall WA. Distinct subcellular localization of different sodium channel alpha and beta subunits in single ventricular myocytes from mouse heart. Circulation 2004;109:1421-1427.
151. Medeiros-Domingo A, Kaku T, Tester DJ, et al. SCN4B-encoded sodium channel beta4 subunit in congenital long-QT syndrome. Circulation 2007;116:134-142.
152. Tan BH, Pundi KN, Van Norstrand DW, et al. Sudden infant death syndrome-associated mutations in the sodium channel beta subunits. Heart Rhythm 2010;7:771-778.
153. Ng D, Johnston JJ, Teer JK, et al. Interpreting secondary cardiac disease variants in an exome cohort. Circ Cardiovasc Genet 2013;6:337-346.
154. Lieve KV, Williams L, Daly A, et al. Results of genetic testing in 855 consecutive unrelated patients referred for long QT syndrome in a clinical laboratory. Genet Test Mol Biomarkers 2013;17:553-561.

SCN5A

155. Gellens ME, George AL, Jr., Chen LQ, et al. Primary structure and functional expression of the human cardiac tetrodotoxin-insensitive voltage-dependent sodium channel. Proc Natl Acad Sci U S A 1992;89:554-558.
156. Wang Q, Shen J, Splawski I, et al. SCN5A mutations associated with an inherited cardiac arrhythmia, long QT syndrome. Cell 1995;80:805-811.
157. Bennett PB, Yazawa K, Makita N, George AL, Jr. Molecular mechanism for an inherited cardiac arrhythmia. Nature 1995;376:683-685.
158. Wang DW, Yazawa K, George AL, Jr., Bennett PB. Characterization of human cardiac Na+ channel mutations in the congenital long QT syndrome. Proc Natl Acad Sci U S A 1996;93:13200-13205.
159. Yamagishi H, Furutani M, Kamisago M, et al. A de novo missense mutation (R1623Q) of the SCN5A gene in a Japanese girl with sporadic long QT sydrome. Mutations in brief no. 140. Online. Hum Mutat 1998;11:481.
160. Benhorin J, Goldmit M, MacCluer JW, et al. Identification of a new SCN5A mutation, D1840G, associated with the long QT syndrome. Mutations in brief no. 153. Online. Hum Mutat 1998;12:72.

161. Kambouris NG, Nuss HB, Johns DC, Tomaselli GF, Marban E, Balser JR. Phenotypic characterization of a novel long-QT syndrome mutation (R1623Q) in the cardiac sodium channel. Circulation 1998;97:640-644.
162. Bezzina C, Veldkamp MW, van Den Berg MP, et al. A single Na(+) channel mutation causing both long-QT and Brugada syndromes. Circ Res 1999;85:1206-1213.
163. Wattanasirichaigoon D, Vesely MR, Duggal P, et al. Sodium channel abnormalities are infrequent in patients with long QT syndrome: identification of two novel SCN5A mutations. Am J Med Genet 1999;86:470-476.
164. Wei J, Wang DW, Alings M, et al. Congenital long-QT syndrome caused by a novel mutation in a conserved acidic domain of the cardiac Na+ channel. Circulation 1999;99:3165-3171.
165. Veldkamp MW, Viswanathan PC, Bezzina C, Baartscheer A, Wilde AA, Balser JR. Two distinct congenital arrhythmias evoked by a multidysfunctional Na(+) channel. Circ Res 2000;86:E91-97.
166. Wedekind H, Smits JP, Schulze-Bahr E, et al. De novo mutation in the SCN5A gene associated with early onset of sudden infant death. Circulation 2001;104:1158-1164.
167. Nuyens D, Stengl M, Dugarmaa S, et al. Abrupt rate accelerations or premature beats cause life-threatening arrhythmias in mice with long-QT3 syndrome. Nat Med 2001;7:1021-1027.
168. Valdivia CR, Ackerman MJ, Tester DJ, et al. A novel SCN5A arrhythmia mutation, M1766L, with expression defect rescued by mexiletine. Cardiovasc Res 2002;55:279-289.
169. Keller DI, Acharfi S, Delacretaz E, et al. A novel mutation in SCN5A, delQKP 1507-1509, causing long QT syndrome: role of Q1507 residue in sodium channel inactivation. J Mol Cell Cardiol 2003;35:1513-1521.
170. Wehrens XH, Rossenbacker T, Jongbloed RJ, et al. A novel mutation L619F in the cardiac Na+ channel SCN5A associated with long-QT syndrome (LQT3): a role for the I-II linker in inactivation gating. Hum Mutat 2003;21:552.
171. Groenewegen WA, Bezzina CR, van Tintelen JP, et al. A novel LQT3 mutation implicates the human cardiac sodium channel domain IVS6 in inactivation kinetics. Cardiovasc Res 2003;57:1072-1078.
172. Chang CC, Acharfi S, Wu MH, et al. A novel SCN5A mutation manifests as a malignant form of long QT syndrome with perinatal onset of tachycardia/bradycardia. Cardiovasc Res 2004;64:268-278.
173. Tian XL, Yong SL, Wan X, et al. Mechanisms by which SCN5A mutation N1325S causes cardiac arrhythmias and sudden death in vivo. Cardiovasc Res 2004;61:256-267.
174. Remme CA, Verkerk AO, Nuyens D, et al. Overlap syndrome of cardiac sodium channel disease in mice carrying the equivalent mutation of human SCN5A-1795insD. Circulation 2006;114:2584-2594.
175. Ruan Y, Liu N, Bloise R, Napolitano C, Priori SG. Gating properties of SCN5A mutations and the response to mexiletine in long-QT syndrome type 3 patients. Circulation 2007;116:1137-1144.
176. Wang DW, Crotti L, Shimizu W, et al. Malignant perinatal variant of long-QT syndrome caused by a profoundly dysfunctional cardiac sodium channel. Circ Arrhythm Electrophysiol 2008;1:370-378.

177. Kapa S, Tester DJ, Salisbury BA, et al. Genetic testing for long-QT syndrome: distinguishing pathogenic mutations from benign variants. Circulation 2009;120:1752-1760.
178. Huang H, Priori SG, Napolitano C, O'Leary ME, Chahine M. Y1767C, a novel SCN5A mutation, induces a persistent Na+ current and potentiates ranolazine inhibition of Nav1.5 channels. Am J Physiol Heart Circ Physiol 2011;300:H288-299.
179. Malan D, Friedrichs S, Fleischmann BK, Sasse P. Cardiomyocytes obtained from induced pluripotent stem cells with long-QT syndrome 3 recapitulate typical disease-specific features in vitro. Circ Res 2011;109:841-847.
180. Carrasco JI, Izquierdo I, Medina P, Arnau MA, Salvador A, Zorio E. Flecainide, a therapeutic option in a patient with long QT syndrome type 3 caused by the heterozygous V411M mutation in the SCN5A gene. Rev Esp Cardiol (Engl Ed) 2012;65:1058-1059.
181. Ma D, Wei H, Zhao Y, et al. Modeling type 3 long QT syndrome with cardiomyocytes derived from patient-specific induced pluripotent stem cells. Int J Cardiol 2013;168:5277-5286.
182. Takahashi K, Shimizu W, Miyake A, Nabeshima T, Nakayashiro M, Ganaha H. High prevalence of the SCN5A E1784K mutation in school children with long QT syndrome living on the Okinawa islands. Circ J 2014;78:1974-1979.
183. Zhang Y, Wang J, Chang S, et al. The SCN5A mutation A1180V is associated with electrocardiographic features of LQT3. Pediatr Cardiol 2014;35:295-300.
184. Wilde AA, Moss AJ, Kaufman ES, et al. Clinical Aspects of Type 3 Long-QT Syndrome: An International Multicenter Study. Circulation 2016;134:872-882.
185. Asadi M, Foo R, Bhuiyan ZA, et al. Genetic analysis of cardiac SCN5A Gene in Iranian patients with hereditary cardiac arrhythmias. Anatol J Cardiol 2016;16:170-174.
186. Ter Bekke RMA, Isaacs A, Barysenka A, et al. Heritability in a SCN5A-mutation founder population with increased female susceptibility to non-nocturnal ventricular tachyarrhythmia and sudden cardiac death. Heart Rhythm 2017;14:1873-1881.

SNTA1

187. Wu G, Ai T, Kim JJ, et al. alpha-1-syntrophin mutation and the long-QT syndrome: a disease of sodium channel disruption. Circ Arrhythm Electrophysiol 2008;1:193-201.
188. Ueda K, Valdivia C, Medeiros-Domingo A, et al. Syntrophin mutation associated with long QT syndrome through activation of the nNOS-SCN5A macromolecular complex. Proc Natl Acad Sci U S A 2008;105:9355-9360.
189. Hu RM, Tan BH, Orland KM, et al. Digenic inheritance novel mutations in SCN5a and SNTA1 increase late I(Na) contributing to LQT syndrome. Am J Physiol Heart Circ Physiol 2013;304:H994-H1001.
190. Choi JI, Wang C, Thomas MJ, Pitt GS. alpha1-Syntrophin Variant Identified in Drug-Induced Long QT Syndrome Increases Late Sodium Current. PLoS One 2016;11:e0152355.

TRDN

191. Altmann HM, Tester DJ, Will ML, et al. Homozygous/Compound Heterozygous Triadin Mutations Associated With Autosomal-Recessive Long-QT Syndrome and Pediatric Sudden Cardiac Arrest: Elucidation of the Triadin Knockout Syndrome. Circulation 2015;131:2051-2060.
192. Walsh MA, Stuart AG, Schlecht HB, James AF, Hancox JC, Newbury-Ecob RA. Compound Heterozygous Triadin Mutation Causing Cardiac Arrest in Two Siblings. Pacing Clin Electrophysiol 2016;39:497-501.
